# Can Plant Extracts Help Prevent Hair Loss or Promote Hair Growth? A Review Comparing Their Therapeutic Efficacies, Phytochemical Components, and Modulatory Targets

**DOI:** 10.3390/molecules29102288

**Published:** 2024-05-13

**Authors:** Joon Yong Choi, Min Young Boo, Yong Chool Boo

**Affiliations:** 1 Department of Biomedical Science, The Graduate School, Kyungpook National University, 680 Gukchaebosang-ro, Jung-gu, Daegu 41944, Republic of Korea; halo134679@knu.ac.kr; 2 BK21 Plus KNU Biomedical Convergence Program, Kyungpook National University, 680 Gukchaebosang-ro, Jung-gu, Daegu 41944, Republic of Korea; 3 Ppeum Clinic Daegu, 39 Dongseong-ro, Jung-gu, Daegu 41937, Republic of Korea; qnalsdud96@gmail.com; 4 Department of Molecular Medicine, School of Medicine, Kyungpook National University, 680 Gukchaebosang-ro, Jung-gu, Daegu 41944, Republic of Korea; 5 Cell and Matrix Research Institute, Kyungpook National University, 680 Gukchaebosang-ro, Jung-gu, Daegu 41944, Republic of Korea; 6 Lapivu Co., Ltd., 115 Dongdeok-ro, Jung-gu, Daegu 41940, Republic of Korea

**Keywords:** alopecia, baldness, natural product, dermal papilla, hair follicle, hair cycle, cell signaling pathway, animal model, clinical study

## Abstract

This narrative review aims to examine the therapeutic potential and mechanism of action of plant extracts in preventing and treating alopecia (baldness). We searched and selected research papers on plant extracts related to hair loss, hair growth, or hair regrowth, and comprehensively compared the therapeutic efficacies, phytochemical components, and modulatory targets of plant extracts. These studies showed that various plant extracts increased the survival and proliferation of dermal papilla cells in vitro, enhanced cell proliferation and hair growth in hair follicles ex vivo, and promoted hair growth or regrowth in animal models in vivo. The hair growth-promoting efficacy of several plant extracts was verified in clinical trials. Some phenolic compounds, terpenes and terpenoids, sulfur-containing compounds, and fatty acids were identified as active compounds contained in plant extracts. The pharmacological effects of plant extracts and their active compounds were associated with the promotion of cell survival, cell proliferation, or cell cycle progression, and the upregulation of several growth factors, such as IGF-1, VEGF, HGF, and KGF (FGF-7), leading to the induction and extension of the anagen phase in the hair cycle. Those effects were also associated with the alleviation of oxidative stress, inflammatory response, cellular senescence, or apoptosis, and the downregulation of male hormones and their receptors, preventing the entry into the telogen phase in the hair cycle. Several active plant extracts and phytochemicals stimulated the signaling pathways mediated by protein kinase B (PKB, also called AKT), extracellular signal-regulated kinases (ERK), Wingless and Int-1 (WNT), or sonic hedgehog (SHH), while suppressing other cell signaling pathways mediated by transforming growth factor (TGF)-β or bone morphogenetic protein (BMP). Thus, well-selected plant extracts and their active compounds can have beneficial effects on hair health. It is proposed that the discovery of phytochemicals targeting the aforementioned cellular events and cell signaling pathways will facilitate the development of new targeted therapies for alopecia.

## 1. Introduction

Hair, a filament-like structure composed of keratin proteins and melanin pigments, grows from the dermis and goes out of the epidermis [1]. Its upper part is called the hair shaft and the lower part is called the hair root [2]. The hair and various cells and matrices around and below it form a mini-organ called a hair follicle [2,3]. The lateral sides of the hair root are surrounded by the inner and outer root sheath cells [4]. The underside of the hair root is bulb-shaped, and the hair root is in contact with the papilla cells of the dermis, which are surrounded by matrix cells (keratinocytes) [5,6]. The capillaries in the subcutaneous tissue beneath the papilla provide the nutrients, oxygen, and growth factors necessary for hair growth. Stem cells reside in the outer root sheath, located in the bulge of the hair follicle [7,8]. Dermal papilla cells release hormones that stimulate the differentiation of stem cells into different cell types via progenitor cells. Matrix cells act as germ cells and differentiate into the inner root sheath and keratin-producing cells. These cells continue dividing, proliferating, differentiating, and keratinizing, leading to hair production and growth. Melanocytes within the layer of matrix cells produce and supply melanin pigments, which are incorporated into the hair.

Hairs contribute to various skin functions, such as physical protection, insulation, sebum dispersal, sensory perception, etc. [9]. Additionally, in human society, hair greatly impacts self-esteem, quality of life, attractiveness, and social interactions [10]. Various factors, such as genetics, immune reactions, hormonal imbalances, inflammation, increased stress, poor nutrition, and medications, can cause hair loss accompanied by anagen to telogen transition [11,12,13,14]. Although hair loss is not a major disease that threatens life or entails serious functional disability, some people are saddened and dissatisfied with hair loss since it affects human appearance [15].

The hair cycle consists of three distinct phases: anagen (growth) phase, catagen (regression, intermediate, or transition) phase, and telogen (resting) phase [7]. The anagen phase lasts 3 to 5 years and more than 80% of human hair is in this phase. The catagen phase lasts about a month and 3% of human hair is in this phase. In the catagen phase, hair growth stops, and the hair bulb recedes toward the surface of the scalp. The telogen phase lasts, on average, 2 to 7 months, and 10 to 20% of human hair is in this phase. In the telogen phase, hairs are loosely attached to the hair follicle while its bulb is dormant. At the end of the telogen phase, when a new hair cycle begins, new hair shafts push out existing hairs, causing them to fall out. This stage is also classified separately as the exogen (shedding) phase [16].

Hair loss types are classified into scarring alopecia, non-scarring alopecia, and structural hair disorders [17]. Scarring alopecia is caused by tissue damage that leads to the irreversible and permanent loss of hair follicles. In non-scarring alopecia, the function of hair follicles is temporally suppressed but may be recoverable using certain treatments, leading to hair regrowth. The fragility of the hair shafts causes structural hair disorders. Non-scarring alopecia includes focal hair loss, diffused hair loss, and patterned hair loss, such as androgenetic alopecia in men (male pattern hair loss), female pattern hair loss, and trichotillomania [18].

Several medicines can treat hair loss in humans [19]. Minoxidil, originally developed as a drug to lower blood pressure by dilating blood vessels, was unexpectedly found to stimulate hair growth, and thus was later developed as a hair growth promoter [20,21,22]. Minoxidil has been described to stimulate cell proliferation, vascular endothelial growth factor (VEGF) expression, and prostaglandin synthesis while inhibiting collagen synthesis in various skin and hair follicle cell types [23]. Finasteride and dutasteride, inhibitors of steroid 5α-reductase enzyme, which converts testosterone into dihydrotestosterone (DHT), were originally developed to treat the symptoms of benign prostatic hyperplasia [24] and are also used to treat male androgenetic alopecia [25]. Finasteride selectively inhibits steroid 5α-reductase type II isozyme and dutasteride inhibits both type I and II isozymes [26]. Various other strategies including cell-based treatments [27] and natural product-based treatments [28] are being attempted to treat hair loss.

Plants have unique survival strategies and synthesize and utilize various metabolites that animals do not have, and these are called phytochemicals [29]. Phytochemicals are broadly classified into phenolic compounds, terpenes/terpenoids, nitrogen-containing compounds, sulfur-containing compounds, etc., and have various physicochemical, biochemical, and biological activities depending on their chemical structures [30]. Plant extracts have been applied to treat human diseases in traditional medicine, and single compounds derived from plants have been developed into medicines or provided a basis for the development of other new drugs [31]. Plant-derived extracts and compounds have been used to protect the skin against environmental factors, such as ultraviolet rays [32] and air pollution [33], and to alleviate several skin conditions, such as inflammation [34] and keloid scar [35]. The biological activity and pharmacological effects of various plant-derived extracts and compounds have also been studied for their potential application in promoting hair health [28,36,37].

Although several medicines already serve good roles in hair loss prevention and hair growth promotion, natural products can provide an alternative option for hair care, offering ease and comfort to people who do not prefer chemically manufactured oral pills or topical agents. The primary purpose of this review is to examine the therapeutic potential of plant extracts in preventing hair loss or promoting hair growth or regrowth. Given the presence of other review papers on similar topics [28,36,37], this review focuses on comparing the therapeutic efficacies, phytochemical components, and modulatory targets of plant extracts evaluated in recent studies. We hope that this review will contribute to understanding the current status and prospects of research in this field and developing new therapeutic strategies for hair loss.

## 2. Methods

We accessed the PubMed database (https://pubmed.ncbi.nlm.nih.gov/, accessed on 30 April 2024) to search for research articles related to the topic of this narrative review. A preliminary literature search using various keywords, such as ‘hair loss’, ‘hair growth’, ‘hair regrowth’, ‘extract’, ‘plant’, ‘herb’, ‘root’, ‘leaf’, ‘leaves’, ‘stem’, and ‘flower’, and Boolean search commands, such as ‘AND’ and ‘OR’, resulted in hundreds of research articles that were too many to be explored in-depth in a single review paper. We refined the search results by limiting the search ranges for some keywords to title words only to select more highly focused studies. We used the following key terms: (hair loss[Title] OR hair growth[Title] OR hair regrowth[Title]) AND extract[Title] AND (plant OR herb OR root OR leaf OR leaves OR stem OR flower). This search identified 57 research articles written in English. Additionally, we accessed the Web of Science (https://www.webofscience.com/, accessed on 30 April 2024) and Google Scholar (https://scholar.google.com/, accessed on 30 April 2024) databases for an additional literature search, identifying 38 more research articles that examined plant extracts including several marine plants. Most identified research articles are cited and explored in the appropriate chapter(s) according to their contents, excluding a few articles that investigated the extracts of animals or fungi (4 articles), or only pure compounds (2 articles).

Chemical structures of phytochemicals, validated by comparing with the information in the PubChem database (https://pubchem.ncbi.nlm.nih.gov/, accessed on 30 April 2024), were drawn using ACD/ChemSketch 12.0 software (ACD/Labs, Toronto, ON, Canada).

## 3. Therapeutic Efficacies of Plant Extracts

### 3.1. Effects of Plant Extracts on Dermal Papilla Cells In Vitro

The fates of dermal papilla cells are closely related to the hair growth cycle. Therefore, the viability and proliferation of dermal papilla cells are useful targets to prevent hair loss and promote hair growth.

Table 1 summarizes the extracts derived from a plant or several plants that have been reported to enhance the proliferation of human follicle dermal papilla cells (HFDPCs) or related cells in vitro. Table 2 summarizes the plant extracts that enhanced cell viability reduced by testosterone or DHT.

In many studies, cell viability or proliferation was measured using colorimetric assays based on the reduction of dyes, such as 3-(4,5-dimethyl thiazol-2-yl)-2,5-diphenyl tetrazolium (MTT), 3-(4,5-dimethyl thiazol-2-yl)-5-(3-carboxymethoxyphenyl)-2-(4-sulpho phenyl)-2H-tetrazolium (MTS), 2-(2-methoxy-4-nitrophenyl)-3-(4-nitrophenyl)-5-(2,4-disulfophenyl)-2H-tetrazolium (WST-1), 2-(2-methoxy-4-nitrophenyl)-3-(4-nitrophenyl)-5-(2,4-disulfophenyl)-2H-tetrazolium (WST-8), and 7-hydroxy-3*H*-phenoxazin-3-one 10-oxide (resazurin), which mainly reflect mitochondrial function. Assays based on the incorporation of [^3^H]-thymidine or bromodeoxyuridine (BrdU) during DNA synthesis in cells were also used to measure cell proliferation in some studies. Ki-67 nuclear protein is associated with ribosomal RNA transcription [69] and its immunostaining has been used to evaluate cell proliferation in some studies.

Table 1 and Table 2 show the effective concentrations of plant extracts that enhanced the proliferation or viability of dermal papilla cells, as reported in previous studies. These data will be helpful in roughly comparing the relative activities of various plant extracts and selecting plant extracts with high application potential. More accurate and reliable information can be obtained through studies that directly measure and compare the activities of various extracts under the same conditions.

It is interesting to observe that male hormones reduced the viability of dermal papilla cells and that several plant extracts restored cell viability [66,67,68]. The camellia (*Camellia japonica*) extract promoted cell proliferation and alleviated the decline in cell viability caused by androgenic hormones [61,67].

### 3.2. Effects of Plant Extracts on Hair Follicles Ex Vivo

In several previous studies, the effects of various plant extracts on hair growth, hair cycle, and proliferation of the associated cells were evaluated in experiments ex vivo using hair follicles obtained from human or animal donors, as summarized in Table 3.

Extracts from various plants, such as *Cucumis melo*, *Orthosiphon stamineus*, and *Panax ginseng*, promoted hair shaft growth in organ-cultured hair follicles [47,63,70]. The extract from *Cucumis melo* promoted the proliferation of keratinocytes in the hair bulb and matrix constituting the hair follicles [70]. Additionally, extracts from some plants, such as *Cucumis melo*, *Houttuynia cordata*, and *Polygonum multiflorum*, prolonged the anagen phase of the hair cycle [49,51,70]. *Brassica oleracea* and *Panax ginseng* extracts restored hair shaft growth and proliferation of constituent cells in hair follicles, respectively, which were suppressed by testosterone or DHT [43,66]. The ex vivo experimental results suggest the therapeutic potential of these plant extracts to improve hair growth.

### 3.3. Effects of Plant Extracts on Hair Growth in Animal Models In Vivo

Table 4 summarizes the effects of various plant extracts on hair growth in animal models. The test substance, animal model, vehicle or formula of the test substance, route and period of administration, measurement items, and comparison data between groups are shown. The list includes the extracts of marine plants, such as *Eucheuma cottonii* [73] and *Sargassum fusiforme* [74]. 

Mice and rats have often been used as animal models to evaluate hair growth whereas rabbits or guinea pigs have rarely been used [107,111,112,115,116]. Many studies have used C57BL/6 mice, which have the advantage of being easy to observe with the naked eye due to their dark fur color. Some studies have used different substrains of C57BL/6 mice, such as C57BL/6N [79], C57BL/6NCrSlc [84], and C57BL/6J [58], although this does not mean that a particular substrain is more suitable for hair growth studies. Animals of different colors also have been used in hair growth research without major problems. Previous studies have used C3H mice with brown fur [38,39,82,84], albino mice with white fur [103,109], and albino Wistar rats or Sprague–Dawley rats with white fur [78,95]. These animal models commonly require hair removal in hair growth research, but athymic BALB/c nude mice with natural hair growth defects do not require hair removal, providing an alternative model [90].

When mice are about 7 weeks old, most of the hair on their skin is synchronized in the telogen phase [117], so removing hair from mice at this age can help reduce inter-individual variation in the hair cycle. Hair removal methods include shaving with clippers or applying a kind of hair-removing solution or product followed by wiping to remove [58]. Some previous studies have developed animal models that mimic hormonal hair loss conditions by topical application or subcutaneous injection of testosterone in mice [68,84] or that mimic menopause conditions by ovariectomy in female Sprague–Dawley rats [95]. The chronic restraint stress model has also been used in hair research [96].

The plant extract has been applied topically in many studies, but it has also been administered via subcutaneous injection [39,42] or oral feeding [68,95]. When applying a test substance topically, it is necessary to optimize the vehicle by considering the solubility of the drug, skin irritation, and skin absorption. Typically, propylene glycol, ethanol (EtOH), glycerin, and water have been used alone or in combination as a vehicle. Test substances were administered once a day in most cases, yet there were also cases where they were administered twice a day or once every few days. Many studies used minoxidil as the positive control, while finasteride has also been used [68].

The entire period of test substance administration after hair removal varied depending on the study, from 2 weeks [68,82] to 7 weeks [43], and the measurement of hair growth often continued until the hairs in the hair removal area had grown to the length of the surrounding area. However, in a study that counted the number of hair follicles per unit skin area or hair shafts per follicle, the test substance was administered for 10 days [77] or 3 months [95]. Overall, the test period can vary depending on the test purpose and measurement items.

Various plant extracts promoted hair growth or alleviated the delay in hair growth caused by androgen hormones in animal models. Some plant extracts promoted telogen-to-anagen conversion in the hair cycle. Therefore, many of these extracts have potential applications in preventing and treating human alopecia.

It is difficult to compare the hair growth-promoting efficacy of plant extracts evaluated separately in different studies. However, suppose individual studies include negative or positive controls or multiple test groups administered various doses of the test substance. In that case, it is possible to interpret the reliability of the experimental results and the relative efficacy of the test substance. It is also necessary to conduct follow-up studies by prioritizing plant extracts that showed relatively strong efficacy in reliable studies compared to positive controls.

### 3.4. Clinical Studies on the Hair Growth Promotion or Suppression Efficacy of Plant Extracts

In clinical trials examining hair loss and hair growth, a combination of instrumental analysis and visual evaluation is used [118,119]. Table 5 summarizes several double-blind, randomized, placebo-controlled trials on human subjects that evaluated the efficacy of a solution, tonic, lotion, cream, or shampoo containing different plant extracts promoting or suppressing hair growth.

Topical application of the products containing *Stryphnodendron adstringens* bark extract and *Curcuma aeruginosa* extract reduced the growth of terminal hairs and axillary hairs, respectively, in women [120,122]. In contrast, topical application of the products containing the extract of *Thuja occidentalis*, *Oryza sativa*, *Curcuma aeruginosa*, *Centipeda minima*, or *Silybum marianum* increased hair density in all human subjects [65,72,118,121,124,125]. Topical application of a product containing herbal mixture extracts also promoted hair growth and reduced hair loss in human subjects [45,123]. These results suggest that plant extracts may have different effects of enhancing or inhibiting hair growth in various body parts depending on their types, contents, and formulas. Therefore, in developing hair care products using plant extracts, multiple factors must be considered to realize the purpose of use. Some plant extracts have been reported to help increase hair density when taken orally [62,126], so research on the route of administration is also needed.

## 4. Phytochemical Components and Active Compounds in Plant Extracts

As shown in Table 6, the main phytochemical components and active compounds of plant extracts have been presented in several studies. In this chapter, we will examine these compounds by dividing them into phenolic compounds, terpenes and terpenoids, sulfur-containing compounds, fatty acids, and other compounds.

### 4.1. Phenolic Compounds

**Figure 1:**
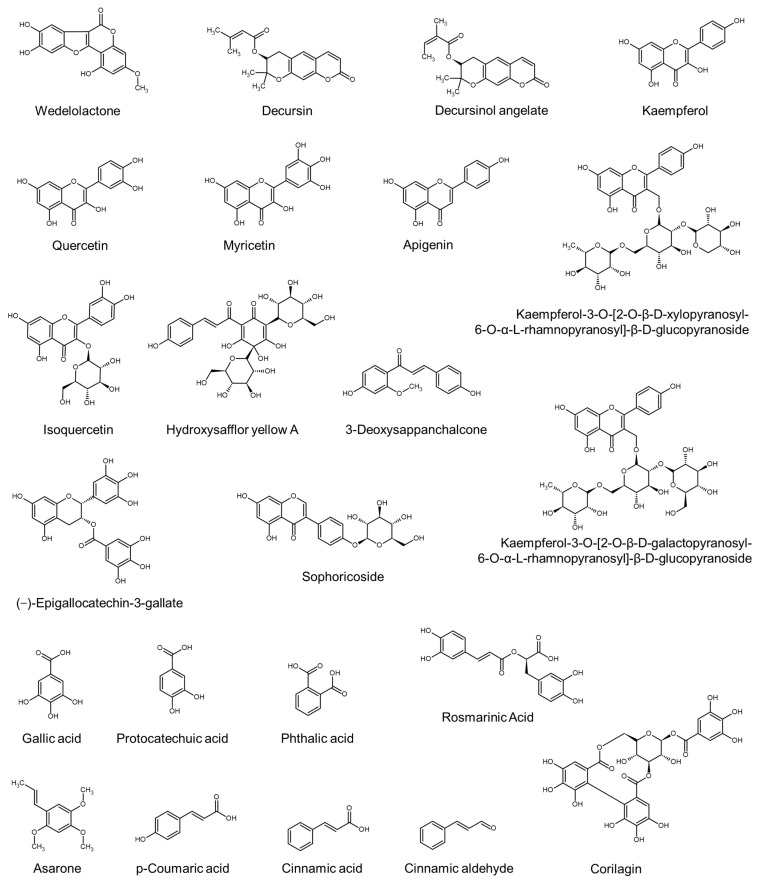
Chemical structures of phenolic compounds.

The chemical structures of some phenolic compounds are shown in Figure 1. The phenolic compounds include coumarins (e.g., weldelolactone, decursin, and decursinol angeleate), phenolic acids (gallic acid, protocatechuic acid, and phthalic acid), phenylpropanoids (e.g., asarone, *p-*coumaric acid, cinnamic acid, cinnamic aldehyde, and rosmarinic acid), flavonoids, and tannins (e.g., corilagin). The flavonoid compounds include flavonols (e.g., kaempferol, quercetin, and myricetin), flavones (e.g., apigenin), isoflavones (e.g., genistein), flavanols (e.g., (−)-epigallocatechin gallate), chalcones (e.g., 3-deoxysappanchalcone), and their glycosides (e.g., isoquercetin, sophoricoside, and hydroxysafflor yellow A).

Extract of *Eclipta alba* contains coumestans including wedelolactone as the main phytochemical components alongside flavonoids, triterpenoid glycosides, triterpenoid saponins, and thiophene derivatives [77]. Extract from *Angelica gigas* contains coumarin compounds, such as decursin and decursinol angelate [98]. The hair growth-promoting effect of decursin was confirmed in male C57/BL6 mice [98]. Decursin reduced the expression of inflammatory cytokines, such as tumor necrosis factor α (TNF-α) and interleukin (IL)-1β, while increasing the expression of anti-inflammatory cytokines IL-4 and IL-13, and an inflammation mediator, high-mobility group box 1 (HMGB1) [98].

Extract from *Thuja orientalis* contains flavonoids, such as kaempferol and isoquercetin [83]. Extract from *Silybum marianum* contains apigenin as the main component [65]. Extracts of *Diospyros kaki*, *Camellia sinensis*, and *Sophora Japonica* contain tannic acids, (−)-epigallocatechin-3-gallate, and sophoricoside (an isoflavone genistein glycoside), respectively [126]. Extract of a herbal mixture (*Urtica urens*, *Urtica dioica*, *Matricaria chamomilla*, *Achillea millefolium*, *Ceratonia siliqua*, and *Equisetum arvense*) contains kaempferol, quercetin, and myricetin [123]. Extract from *Camellia japonica* contains gallic acid, protocatechuic acid, kaempferol-3-*O*-[2-*O*-β-D-galactopyranosyl-6-*O*-α-L-rhamno pyranosyl]-β-D-glucopyranoside, and kaempferol-3-*O*-[2-*O*-β-D-xylopyranosyl-6-*O*-α-L-rhamnopyranosyl]-β-D-glucopyranoside [61,67]. Extract from *Carthamus tinctorius* contains 212.00 ± 17.56 mg g^−1^ of hydroxysafflor yellow A, a single chalcone glycoside, as the main phytochemical component [41]. Extract of *Caesalpinia sappan* contains 3-deoxysappanchalcone [124].

Hot water extract of a herbal mixture (*Acorus calamus*, *Morus alba*, *Glycyrrhiza uralensis*, *Pinus densiflora*, *Sophora angustifolia*, *Ligusticum chuanxiong*, and *Angelica giga*) contains phenylpropanoid compounds, such as asarone (from *Acorus calamus*) and *p*-coumaric acid (from *Pinus densiflora*), as the main components [91]. Extract of *Cinnamomum osmophloeum* contains cinnamic aldehyde and cinnamic acid, which are also phenylpropanoid compounds [48].

Extract of *Geranium sibiricum* contains gallic acid and corilagin (an ellagitannin) [46]. Extracts of *Perilla frutescens* and *Lycopus lucidus* contain rosmarinic acid as the main component [59,71], and extract of *Allium ascalonicum* contains rosmarinic acid, *p-*coumaric acid, and quercetin [57]. Rosmarinic acid was shown to attenuate cell death caused by testosterone and promote VEGF gene expression in cells [57,59,71]. Of the various phytochemical components in *Leea indica* extract, phthalic acid and other several compounds have been proposed as potential inhibitors of prostaglandin D_2_ synthase based on in silico ligand binding analysis [103].

### 4.2. Terpenes and Terpenoids

**Figure 2:**
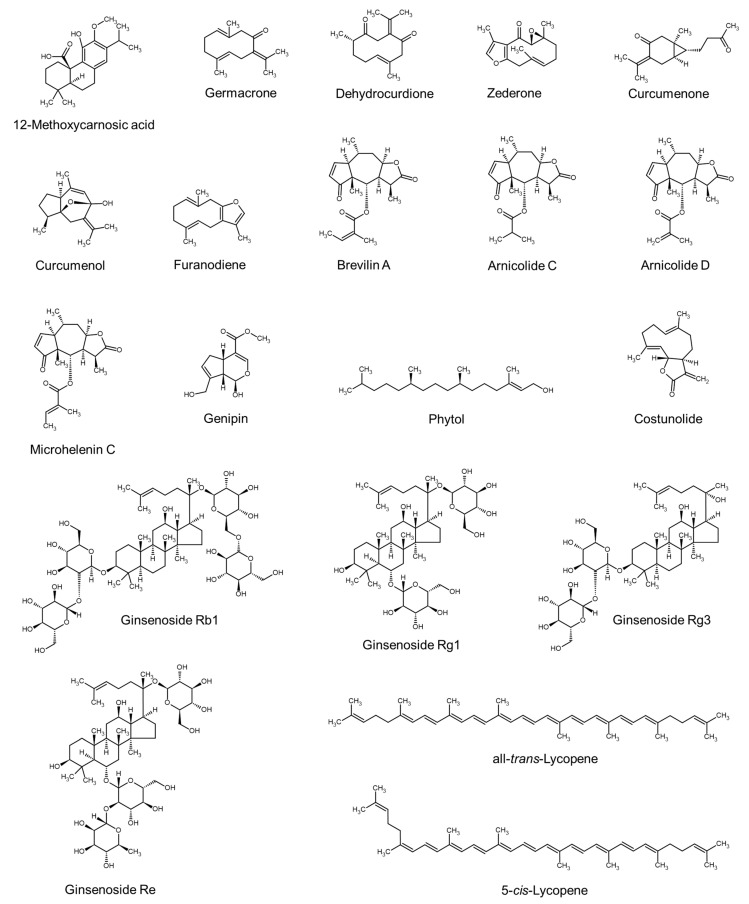
Chemical structures of terpenes and terpenoids.

The chemical structures of some terpenes and terpenoids are shown in Figure 2. Terpenes are composed of isoprene (C_5_H_8_) units and are classified into monoterpenes (C_10_H_16_), sesquiterpenes (C_15_H_24_), diterpenes (C_20_H_32_), triterpenes (C_30_H_48_), and tetraterpenes (C_40_H_64_). Terpenoids are structurally similar to terpenes but have functional groups with heteroatoms such as oxygen.

From the extract of *Rosmarinus officinalis*, 12-methoxycarnosic acid, a diterpenoid, was isolated as an active compound and this compound enhanced the proliferation of cultured LNCaP cells [84]. Extract of *Curcuma aeruginosa* contains high amounts of germacrone and other volatile sesquiterpenoids, such as dehydrocurdione, zederone, cucumenone, curcumenol, and furanodiene [122]. Extract of *Centipeda minima* contains high amounts of brevilin A and several other sesquiterpene lactones, such as arnicolide C, arnicolide D, and microhelenin C [125]. Extract of *Stachytarpheta jamaicensi* contains genipin (a monoterpene iridoid compound, phytol (a hydrogenated diterpene alcohol), and fatty acids (e.g., α-linolenic acid, palmitic acid, and tridecanoic acid) [89]. Extract of *Inula helenium* contains costunolide, a sesquiterpene lactone [124].

*Panax ginseng* extracts contain unique triterpenoid saponins, such as ginsenosides Rb1, Rg1, Rg3, and Re [43,63]. Testosterone suppressed the proliferation of hair matrix keratinocytes in hair follicle explants while upregulating androgen receptors in cultured hDPCs, and all these changes were inhibited by ginsenosides Rb1 and Rg3 [43]. Ginsenosides Rb1, Rg1, and Re enhanced the proliferation of iDPCs while decreasing the mRNA level of BMP4 [63]. A purified extract of *Lycopersicon esculentum* contains high amounts of tetraterpene carotenoids, such as all-*trans*-lycopene and 5-*cis*-lycopene, which are the main active components associated with hair growth-promoting effects [85].

### 4.3. Sulfur-Containing Compounds, Fatty Acids, and Other Compounds

**Figure 3:**
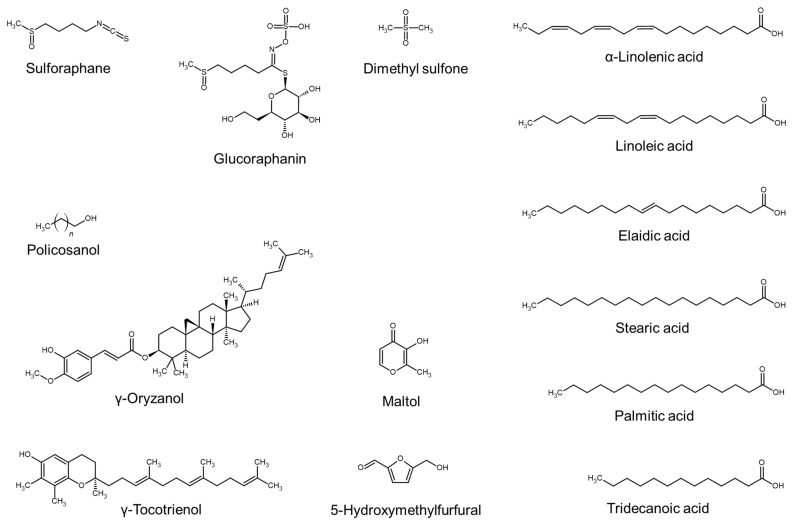
Chemical structures of sulfur-containing compounds, fatty acids, and other compounds.

The chemical structures of some sulfur-containing compounds, fatty acids, and other miscellaneous compounds are shown in Figure 3.

Extract of *Brassica oleracea* contains sulforaphane and glucoraphanin (a glucosinolate of sulforaphane) [66]. These components promoted hair shaft growth in hair follicles derived from C57/BL6 mice [66]. Dimethyl sulfone has been isolated as an active compound from extract of *Blumea eriantha*, and the isolated compound increased the length of the hair follicle [109].

A fat-soluble extract of *Boehmeria nipononivea* contains large amounts of α-linolenic acid, linoleic acid, and palmitic acid [75]. When comparing the hair growth-promoting effects of various fatty acids in C57/BL6 mice, α-linolenic acid, elaidic acid, and stearic acid were more effective than others [75].

Extract of *Oryza sativa* brans contains various primary and secondary metabolites, such as linoleic acid, policosanol, γ-oryzanol, and γ-tocotrienol [86]. As a result of testing hair growth-promoting effects in C57/BL6 mice, linoleic acid was evaluated to be more effective than other compounds [86]. Extract of *Punica granatum* contains maltol, 5-hydroxymethylfurfural, and other volatile phytoconstituents [104].

## 5. Modulatory Targets of Plant Extracts

### 5.1. Antioxidant, Anti-Inflammatory, and Anti-Senescence Effects of Plant Extracts

Oxidative stress induced by external and internal factors is expressed as an increase in prooxidants, a decrease in antioxidants, and an increase in oxidative damage [127]. It acts as a causative mechanism disrupting the homeostasis of the skin, scalp, and hair [128,129]. Reactive oxygen species (ROS), which mediate oxidative stress, can cause an inflammatory response and cellular senescence, hindering hair growth and triggering hair loss [129,130]. Ultraviolet rays and air pollution have been shown to cause oxidative stress in dermal papilla cells and increase cell death [131,132]. Various types of antioxidants have been studied as a defense for scalp and hair [133,134].

As summarized in Table 7, some plant extracts scavenged free radicals in vitro [42,46,55,61,135], reduced intracellular ROS levels [61,65], or enhanced the viability of cells exposed to hydrogen peroxide (H_2_O_2_) [61,65] or 2,2′-azobis (2-amidinopropane) dihydrochloride (AAPH) radical [54]. Some extracts alleviated inflammatory response determined by the expression levels of inflammatory cytokines, such as tumor necrosis factor-alpha (TNF-α), interleukin (IL)-1β, and IL-6 [59,65,67,98], or cellular senescence determined by the expression level of senescence-associated β-galactosidase (SA-β-gal) [61,65,67] in cells stimulated with phorbol-12-myristate 13-acetate (PMA) plus calcium ionophore A23187 [50], H_2_O_2_ [59,61,65], or androgen [67]. The anti-inflammatory effects of extracts of *Angelica gigas* and *Pinus thunbergii* were shown by the reduced levels of pro-inflammatory cytokines (TNF-α and IL-1β) and increased levels of anti-inflammatory cytokines (IL-4 and IL-13) in the dorsal skin of mice [50,108]. In silico molecular docking analysis of phytochemical components of *Leea indica* resulted in the identification of several compounds with high ligand efficiencies towards prostaglandin D_2_ synthase, implicating their potential anti-inflammatory activity [103].

### 5.2. Effects of Plant Extracts on the Apoptotic Cell Death Pathway

Apoptosis is a type of programmed cell death that is executed to remove unnecessary, unhealthy, or unrecoverable cells. In its intrinsic mitochondria-dependent pathway, the ratios of proapoptotic members (e.g., BCL-2-associated X protein (BAX), Bcl-2 homologous antagonist/killer (BAK), and BCL-2 associated agonist of cell death (BAD)) to antiapoptotic members (e.g., B-cell lymphoma 2 (BCL-2), B-cell lymphoma-extra-large (BCL-xL), and myeloid cell leukemia 2 (MCL-2)) of the BCL-2 family increase [136,137]. Incorporating dimers of proapoptotic members into the mitochondrial membrane makes it leaky. Then, cytochrome C is released from the mitochondria and binds to apoptotic protease-activating factor 1 (APAF-1) in the cytoplasm to recruit caspase 9, which in turn activates caspase 3, 6, 7 (called executioner caspases), and other proteases involved in the degradation of cellular components. The extrinsic receptor-dependent apoptosis pathway is mediated by death receptors, such as tumor necrosis factor receptor 1 (TNFR-1) and FAS, and an adaptor, FAS-associated protein with death domain (FADD) [138,139]. The activated receptor and adaptor cooperatively recruit caspase 8, which in turn activates executioner caspases.

As summarized in Table 8, several studies have reported that extracts from several plants, including *Panax ginseng*, *Houttuynia cordata*, and *Camellia japonica*, increased the mRNA or protein level of antiapoptotic BCL-2 [44,49,51,52,67] while decreasing that of proapoptotic BAX [44,49,50,66,67] or BAD [51]. 

### 5.3. Effects of Plant Extracts on Male Hormones

Table 9 shows the effects of some plant extracts on the expression of male hormones and their receptors in cells and animals. It is recognized that an increase in male hormones is highly correlated with hair loss [140] and studies have reported the effects of plant extracts on the expression of male hormones and their receptors in cell and animal models [43,51,62,67,78]. Steroid 5α-reductase type II catalyzes the transformation of testosterone to DHT in cells, and its inhibitor can have therapeutic potential in treating male pattern hair loss [141]. Extracts of several plants and a herbal mixture have been shown to reduce the expression level of steroid 5α-reductase type II in cells [50,53,57,62,67,110,135]. Further, *Sophora flavescens* and *Rosmarinus officinalis* extracts have been shown to inhibit the catalytic activity of steroid 5α-reductase type II in vitro [61,76,84].

### 5.4. Effects of Plant Extracts on Cell Cycle

The cell cycle consists of the gap (G) 1 phase, synthesis (S) phase, G2 phase, mitosis (M) phase, and G0 phase. In the G1 phase, retinoblastoma (Rb) protein sequesters E2F transcription factors and arrests the cell cycle, yet when Rb is hyper-phosphorylated, it releases E2F and the cell cycle enters the S phase [142]. p53 induces the transcription of p21^CIP1^ that inhibits CDK-mediated hyper-phosphorylation of Rb, stabilizing the Rb/E2F complex and causing cell cycle arrest [142]. p16^INK4^ inhibits CDK4 activity and reduces Rb phosphorylation, suppressing cell cycle progression [143].

Table 10 shows several plant extracts that promoted the cell cycle in HFDPCs. The extracts of *Erica multiflora* and *Camellia japonica* increased the percentage of cells in the S or G2/M phase [39,58]. *Houttuynia cordata* and *Camellia japonica* extracts induced the cell cycle G1‒S phase transition by upregulating CDK4 or downregulating p16^INK4^ or p53 [49,67].

### 5.5. Effects of Plant Extracts on the Expression Levels of Growth Factors

As reported in many previous studies, various growth factors, such as insulin-like growth factor (IGF) [144], VEGF [145], hepatocyte growth factor (HGF) [146], and keratinocyte growth factor (KGF) (also called fibroblast growth factor 7, FGF-7) [147], can affect dermal papilla cell physiology or hair growth.

As summarized in Table 11, various plant extracts have been reported to affect the mRNA or protein levels of several growth factors in HFDPCs and animal models. Plant extracts promoting cell proliferation or hair growth generally increased IGF-1, VEGF, HGF, and KGF (FGF-7) levels, with some exceptions.

### 5.6. Effects of Plant Extracts on the AKT and Mitogen-Activated Protein Kinase (MAPK) Signaling Pathways

The activation of phosphoinositide 3-kinases (PI3Ks) and the subsequent phosphorylation and activation of protein kinase B (PKB, also called AKT) by 3-phosphoinositide-dependent kinase 1 (PDK1) or other protein kinases promote cell cycle progression and enhance cell survival [150]. AKT-mediated phosphorylation (inactivation) of glycogen synthase kinase 3 beta (GSK3β) prevents phosphorylation and degradation of cyclin D1, promoting G1‒S phase transition [151]. AKT can inhibit apoptosis by phosphorylating and inactivating several proapoptotic proteins, such as BAD and caspase 9 [152].

Mitogen-activated protein kinases (MAPKs) comprising extracellular signal-regulated kinase (ERK), c-Jun N-terminal kinase (JNK), and p38 MAPK play a critical role in cell physiology [153]. An MAPK cascade is defined as a sequential activation of MAPK kinase kinases (e.g., Raf-1), MAPK kinases (e.g., MEK1 and MEK2), and MAPKs (e.g., ERK1 and ERK2) [154]. The activation of the Raf-1/MEK/ERK pathway leads to the transactivation of target gene expression involved in cell proliferation and other cell functions [155].

Table 12 shows the effect of plant extracts on several protein kinases and protein factors involved in controlling cell fates, such as cell survival, proliferation, and death. Plant extracts derived from *Panax ginseng*, *Rumex japonicas*, *Houttuynia cordata*, *Salvia plebeian*, *Eremochloa ophiuroides*, and *Camellia japonica* stimulated the phosphorylation (activation) of AKT in HFDPCs [43,44,49,52,56,58]. The phosphorylation (activation) of ERK was stimulated by extract from *Panax ginseng*, *Rumex japonicas*, *Houttuynia cordata*, *Salvia plebeian*, *Camellia japonica*, or *Centipeda minima* [43,44,49,52,58,149], and a herbal formula [54]. There are few studies on the phosphorylation (activation) of JNK and p38 MAPK in association with the hair growth-promoting effects of plant extracts [44,149].

### 5.7. Effects of Plant Extracts on the Wingless and Int-1 (WNT) Signaling Pathways

The canonical and non-canonical WNT signaling pathways are involved in regulating cell proliferation, polarity, or migration [156]. In the canonical WNT pathway mediated by β-catenin, the stability of β-catenin is negatively regulated by its phosphorylation at multiple sites by several protein kinases, such as casein kinase 1 (CK1) and GSK3β [156]. When WNT signaling is activated, GSK3β is inactivated through phosphorylation by several protein kinases, such as AKT, or other mechanisms. Then, β-catenin that has avoided proteasomal degradation enters the nucleus, where it acts as a transcriptional coactivator, interacting with several transcription factors, such as lymphoid enhancer-binding factor 1 (LEF1), and regulates the transcription of various target genes, including cyclin D1 and c-Myc [157]. The target genes also include dickkopf 1 (DKK1), which inhibits the WNT pathway in a negative feedback loop [158]. The DKK1 expression level is associated with hair loss; thus, DKK1 inhibition represents an attractive strategy to promote hair growth in androgenetic alopecia [159,160].

Table 13 summarizes the effects of plant extracts on the WNT signaling pathways involved in cell differentiation. The extracts of several plants have been shown to increase the expression of WNTs [61,64,74,99,149] or decrease the expression of DKK1 [51,61,64,72,110]. Several plant extracts have been shown to increase the phosphorylation (inactivation) of GSK3β, upregulating β-catenin levels [44,52,56,149]. Several other extracts also upregulated β-catenin levels [40,57,64,68,79,83,99]. *Gynostemma pentaphyllum* extract also upregulated LEF1 [64]. The extracts of *Mangifera indica*, *Camellia japonica*, and *Terminalia bellirica* increased the expression of downstream targets of the WNT pathway, such as c-Myc and cyclin D1 [61,68,110].

### 5.8. Effects of Plant Extracts on the Sonic Hedgehog (SHH) Signaling Pathways

Hedgehog ligands, including sonic hedgehog (SHH), desert hedgehog (DHH), and Indian hedgehog (IHH), are paracrine signaling factors that mediate cell-to-cell communication [161]. The SHH signaling pathway is involved in regulating hair follicle morphogenesis [162]. The interaction between SHH and the transmembrane protein patched (PTC) triggers the release of smoothened (SMO) from suppressing PTC, which leads to the dissociation of glioma-associated oncogene transcription factor (GLI) from a cytosolic complex [163]. GLI proteins enter the nucleus and act as transcription factors regulating the expression of target genes [164].

Table 14 summarizes plant extracts that affected the SHH signaling pathway. Several plant extracts increased SHH protein levels in hair follicles in animal models. The extract of *Allium ascalonicum* and *Coffea arabica* promoted gene expression of SHH, SMO, and GLI1 at the cellular level [57,135].

### 5.9. Effects of Plant Extracts on the Transforming Growth Factor (TGF)-β and Bone Morphogenetic Protein (BMP) Signaling Pathways

TGF-βs and BMPs are members of the TGF-β superfamily. In the canonical TGF-β signaling pathway, binding of TGF-βs to their receptors induces the phosphorylation of small mothers against decapentaplegic (SMAD) 2 and SMAD3 (called receptor-regulated SMADs or R-SMADs) followed by the formation of a trimeric complex with SMAD4 (called a common partner SMAD or co-SMAD), which enters the nucleus and induces the transcription of target genes [165]. The target genes include SMAD7 (called an inhibitory SMAD or I-SMAD), which blocks TGF-β signaling in a negative feedback loop [166]. In the canonical BMP signaling pathway, SMADs 1, 5, and 8 act as R-SMADs, and SMAD 6 acts as an I-SMAD, whereas SAMD4 acts as a co-SMAD [167]. TGFs and BMPs can also trigger the non-canonical signaling pathways mediated by multiple protein kinases independently of SMADs [167,168]. TGF-βs and BMPs are known to negatively affect hair growth by suppressing hair follicle function and causing hair cycle progression into the telogen phase [169,170].

Table 15 summarizes plant extracts that affect the TGF-β and BMP signaling pathways. Many plant extracts decreased the expression of TGF-β1, TGF-β2, BMP4, SMAD2, and SMAD3 in cell and animal models. Exceptionally, the expression of TGF-β2 was increased by *Cinnamomum osmophloeum* extract [48].

## 6. Discussion

Research has been actively conducted to develop effective and safe treatments for human hair loss using natural products, especially plant-based materials. As explained in the previous sections, the hair growth-promoting potential of plant extracts has been supported in many in vitro experiments using cells (Table 1 and Table 2), ex vivo experiments using hair follicle explants (Table 3), in vivo experiments using mice or rats (Table 4), and clinical trials in humans (Table 5). Experimental groups treated with certain plant extracts had cell proliferation and hair growth significantly higher than negative control groups and comparable to positive control groups treated with minoxidil or finasteride. These results suggest that a beneficial effect on hair growth is expected when plant extracts are administered appropriately.

While hair follicles are mini-organs in which several types of cells interact and cooperate to produce and grow hair, many studies have evaluated the effects of test substances using single-cell models in which only specific cells, such as dermal papilla cells, are cultured (Table 1 and Table 2). Considering that interactions between various constituent cells are important for the function of hair follicles, it is necessary to develop technologies for co-culturing multiple cells or three-dimensional cultures, and further artificially creating hair follicles. Ex vivo experiments using excised hair follicles help to overcome some of the limitations of cell models, and the effect of test substances on hair growth has been successfully evaluated in several ex vivo studies (Table 3). However, there are limitations in the supply of human tissue.

Various animal models have been used for primary efficacy testing of plant extracts (Table 4). Animal hair removal models have been most often used in hair growth research although these models have the disadvantage of having little similarity to natural human hair loss. It is worth noting that several plant extracts showed hair growth promotion efficacy equivalent to or higher than minoxidil, a positive control. These include extracts from *Rumex japonicus* [44], *Cucumis melo* [70], *Perilla frutescens* [71], *Leea indica* [103], *Blumea eriantha* [109], etc.

Animal models in which hair removal is combined with male hormone administration [68,71,84] or ovariectomy [95] have high physiological relevance as models of androgenetic alopecia in men and postmenopausal alopecia in women, respectively. Extracts of *Terminalia bellirica*, *Perilla frutescens*, and *Rosmarinus officinalis* recovered hair growth suppressed by testosterone or DHT [68,71,84]. Extract of *Ribes nigrum* promoted hair growth in ovariectomized female Sprague–Dawley rats [95].

Athymic animals with a congenital tendency for hair loss provide a model for natural hair loss without needing hair removal [90]. In a study using male athymic BALB/c nude mice, extract of *Chrysanthemum zawadskii* promoted hair growth more effectively than extract of *Polygonum multiflorum* [90]. Examining which type of human hair loss is most similar to an animal model is necessary, since it increases the utility of the animal model in hair growth research. An animal model in which hair loss is induced by spatially confined stress may be utilized in studying similar stress-induced alopecia in humans [96].

Although many extracts have shown high potential for hair growth-promoting effects in animal models, only a few have advanced to the level of clinical trials (Table 5). We do not take any position supporting or disputing previously reported clinical trial results. Currently, no matter what the purpose of the use or the route of administration, we do not recommend the human application of any plant extract without its prior confirmed safety. Expansion of clinical trials is necessary to verify the effectiveness and safety of the final product containing plant extracts.

Plants were often extracted using hot water or various organic solvents, such as methanol (MeOH), EtOH, acetone [75], ethyl acetate [85], and n-hexane [122]. Supercritical CO_2_ extraction [85,86], cold vacuum extraction [113], and emulsion-assisted extraction methods [125] have also been used to prepare a special type of plant extract. Solvent partition [71,87,103] and chromatography [71,109] have been used to partially purify or isolate pure active compounds from a crude plant extract. Plant extracts have been formulated in a solution [45], tonic [121,125], lotion [122], cream [120], shampoo [65,123], or nanoparticles [102,171] for topical application. Tablets and other types of food products have also been manufactured for oral administration [62,126]. The improvement in quality control and extraction and purification methods to increase the content of active ingredients in plant extracts and the development of optimized formulas and drug carriers to improve the biological availability and delivery of active compounds to the point of action are needed to prompt the development of effective hair care products using plant extracts.

The biological activity of plant extracts enhancing cell proliferation or hair growth has been attributed to their main phytochemical components (Table 6), such as phenolic compounds (Figure 1), terpenes and terpenoids (Figure 2), sulfur-containing compounds, fatty acids, and other compounds (Figure 3). In some studies, the biological activity of single active compounds has been verified at the cellular level or in vivo. Representative examples of compounds with proven activity include decursin [98], rosmarinic acid [57,59,71], 12-methoxycarnosic acid [84], ginsenosides [43,63], sulforaphane, glucoraphanin [66], dimethyl sulfone [109], α-linolenic acid [75], and linoleic acid [86]. The experimental evidence accumulated so far is insufficient to derive the structure–activity relationship, and we look forward to additional research on this task for optimized drug discovery.

**Figure 4:**
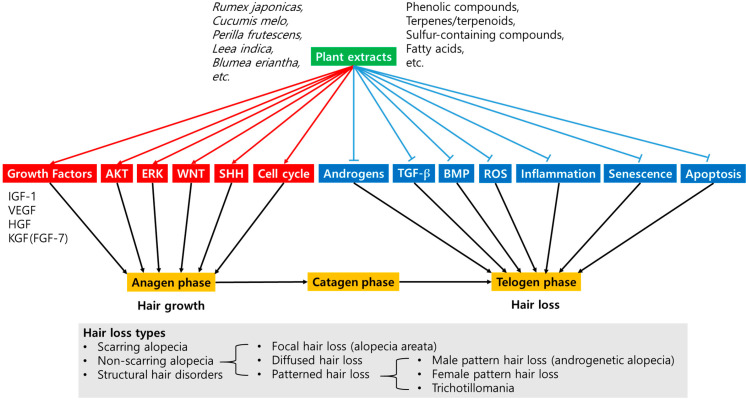
The modulatory targets of plant extracts for promoting hair growth and preventing hair loss. Several plant extracts containing various active phytochemicals can initiate or extend the anagen phase of the hair cycle by stimulating the expression of several growth factors; the AKT, ERK, WNT, and SHH signaling pathways; or inducing the cell cycle progression. Some plant extracts can prevent entry into the telogen phase of the hair cycle by inhibiting androgen expression and the TGF-β and BMP signaling pathways or alleviating ROS-mediated oxidative stress, inflammatory response, cellular senescence, and apoptosis. Plant extracts with different mechanisms of action can show differentiated efficacy according to the type of hair loss with different etiology. Black arrows indicate the hair cycle progression associated with hair growth and loss. Sharp red arrows indicate upregulation, stimulation, or promotion, and blunted blue arrows indicate downregulation, inhibition, or suppression by plant extracts.

Several plant extracts have been shown to prevent alopecia by inducing or prolonging the anagen phase of the hair cycle and inhibiting entry into the telogen phase (Table 3, Table 4 and Table 5). The pharmacological effects of plant extracts that induced and extended the anagen phase in the hair cycle could be associated with the promotion of cell proliferation (Table 1), cell survival (Table 2), or cell cycle progression (Table 10); the upregulation of several growth factors, such as IGF-1, VEGF, HGF, and KGF (FGF-7) (Table 11); and the stimulation of several cell signaling pathways mediated by AKT, ERK, WNT, or SHH (Table 12, Table 13 and Table 14). In addition, the pharmacological effects of plant extracts that prevented the entry into the telogen phase in the hair cycle could be attributed to the alleviation of oxidative stress, inflammatory response, cellular senescence (Table 7), or apoptosis (Table 8); the downregulation of male hormones and their receptors (Table 9); and the suppression of several cell signaling pathways mediated by TGF-β or BMP (Table 15). These findings suggest a potential mechanism of action of plant extracts in promoting hair growth and preventing hair loss, which is schematized in Figure 4.

Because the hair cycle depends on the health and function of various cells in the hair follicles, which are in turn affected by multiple physiological factors, such as hormones and stresses [2,14,172,173,174], it is necessary to analyze in detail the etiology and pathology of alopecia for each patient and develop a customized treatment strategy accordingly. To achieve this, effective medications targeting specific cellular events and cell signaling pathways involved in hair growth and loss are needed. Exploration of plant-based natural products against these modulatory targets will provide a promising opportunity to discover natural remedies or lead compounds for targeted therapies for different types of hair loss.

Overall, research in this field has not only expanded the list of plant extracts and phytochemicals with the potential to promote hair health but has also deepened our understanding of their mechanisms of action. However, there are not many studies that comprehensively explore pharmacological effects, active compounds, and molecular targets of the plant extracts. More integrated and expanded research that reflects the latest knowledge presented in this review is needed to promote the development of improved treatments for alopecia.

## 7. Conclusions

Accumulated evidence from in vitro, in vivo, and clinical studies suggests that several plant extracts and phytochemicals can help prevent hair loss or promote hair growth and regrowth. Well-selected plant extracts can provide additional or alternative hair loss treatment options to people reluctant to use medicines. In addition, the active compounds can serve as lead compounds for new drug discovery and development. Their effects on the hair cycle were associated with the modulation of cell proliferation, cell survival, cell cycle progression, growth factors, hormones, oxidative stress, inflammatory response, cellular senescence, apoptosis, and several cell signaling pathways mediated by AKT, ERK, WNT, SHH, TGF-β, or BMP. Therefore, it is proposed that the discovery of phytochemicals modulating these targets will lead to the development of new targeted therapies for alopecia.

## Figures and Tables

**Table 1:** Effects of plant extracts on the proliferation of dermal papilla cells in vitro.

Plant Extracts	Cell Types	Assays	Effective Concentrations *	Literature
Ethanol (EtOH) extract of roots of *Asiasarum heterotropoides* (or *Asiasarum sieboldi*)	Human follicle dermal papilla cells (HFDPCs)	[3 H]-thymidine incorporation	0.1 μg mL^−1^	Rho et al., 2005 [38]
70% EtOH extract of *Erica multiflora*	HFDPCs	3-(4,5-Dimethyl thiazol-2-yl)-2,5-diphenyl tetrazolium (MTT) reduction	500 and 5000 μg mL^−1^	Kawano et al., 2009 [39]
Water extract of tubers of *Aconiti Ciliare*	Human immortalized dermal papilla cells (iDPCs)	2-(2-Methoxy-4-nitrophenyl)-3-(4-nitrophenyl)-5-(2,4-disulfophenyl)-2H-tetrazolium (WST-1) reduction	5, 10, and 20 μg mL^−1^	Park et al., 2012 [40]
50% EtOH extract of florets of *Carthamus tinctorius*	HFDPCs	MTT reduction	5–1250 μg mL^−1^	Junlatat and Sripanidkulchai, 2014 [41]
50% methanol (MeOH) extract of *Platycarya strobilacea*	HFDPCs	CCK-8 assay using 2-(2-Methoxy-4-nitrophenyl)-3-(4-nitrophenyl)-5-(2,4-disulfophenyl)-2H-tetrazolium (WST-8) reduction	9.8, 19.5, 39.1, and 156.3 μg mL^−1^	Kim et al., 2014 [42]
Extract of red ginseng (*Panax ginseng*)	HFDPCs	CCK-8 assay	300 μg mL^−1^	Park et al., 2015 [43]
95% EtOH extract of roots of *Rumex japonicus*	HFDPCs	MTT reduction	5, 10, 50, and 100 μg mL^−1^	Lee et al., 2016 [44]
DA-5512 formula (EtOH extract of herbal mixture: *Thea sinensis*, *Emblica officinalis*, *Pinus densiflora*, *Pueraria thunbergiana*, *Tribulus terrestris*, and *Zingiber officinale*)	HFDPCs	Ki-67 staining	100 μg mL^−1^	Yu et al., 2017 [45]
MeOH extract of *Geranium sibiricum*	HFDPCs	CCK-8 (WST-8) reduction	19.5 μg mL^−1^	Boisvert et al., 2017 [46]
Extract of *Orthosiphon stamineus*	HFDPCs	PrestoBlue assay using resazurin reduction	25, 50, 125, and 250 μg mL^−1^	Somsukskul et al., 2017 [47]
Water extract of *Cinnamomum osmophloeum*	HFDPCs	MTT reduction	5000 μg mL^−1^	Wen et al., 2018 [48]
50% EtOH extract *Houttuynia cordata*	HFDPCs	Bromodeoxyuridine (BrdU) incorporation	20 and 50 μg mL^−1^	Kim et al., 2019 [49]
RE-ORGA (hot water extract of herbal mixture: *Panax ginseng*, *Glycine max*, *Houttuynia cordata*, *Lycium chinense*, *Glycyrrhiza uralensis*, *Citrus unshiu*, *Zizyphus jujuba*, *Perilla frutescens*, *Camellia sinensis*, and *Cynanchum wilfordii*)	HFDPCs	CCK-8 assay	10,000, 50,000, and 100,000 μg mL^−1^	Kang et al., 2019 [50]
50% EtOH extract of *Polygonum multiflorum*	HFDPCs	CCK-8 assay	10 and 100 μg mL^−1^	Shin et al., 2020 [51]
MeOH extract of *Salvia plebeia*	HFDPCs	CCK-8 assay	15.6, 31.3, and 62.5 μg mL^−1^	Jin et al., 2020 [52]
50% EtOH extract of *Plumbago zeylanica*	HFDPCs	Cell counting	0.2 μg mL^−1^	Yamada et al., 2020 [53]
Phyllotex™ (a herbal formula: *Euterpe oleracea*, *Olea europea*, *Tabebuia impetiginosa*, and *Coffea Arabica*)	HFDPCs	MTT reduction	60–2000 μg mL^−1^	Serruya and Maor, 2021 [54]
50% MeOH extract of lotus (*Nelumbo nucifera*) seeds	HFDPCs	CCK-8 assay	31.25, 62.5, 125, and 250 μg mL^−1^	Park et al., 2021 [55]
80% MeOH extract of centipedegrass (*Eremochloa ophiuroides*)	HFDPCs	MTT reduction	6.2, 12.5, 25, and 50 μg mL^−1^	Ramadhani et al., 2022 [56]
MeOH extract of shallot (*Allium ascalonicum*)	HFDPCs	Sulforhodamine B (SRB) assay	100 μg mL^−1^	Ruksiriwanich et al., 2022 [57]
60% EtOH extract of *Camellia japonica* seed cakes	HFDPCs	MTT reduction	20 μg mL^−1^	Wang et al., 2022 [58]
Hot water extract of *Lycopus lucidus*	HFDPCs	CCK-8 assay	50 μg mL^−1^	Lee et al., 2022 [59]
Hot water extract of mangosteen (*Garcinia mangostana*) pericarps	HFDPCs	WST-1 reduction	62.5, 125, 250, and 500 μg mL^−1^	Tan et al., 2022 [60]
70% EtOH extract of fruit shells of *Camellia japonica*	HFDPCs	Ki-67 staining	10 and 50 μg mL^−1^	You et al., 2023 [61]
Water extract of banana (*Musa paradisiaca*) flowers	HFDPCs	MTT reduction	62.5 and 125 μg mL^−1^	Liang et al., 2023 [62]
20% EtOH extract of *Panax ginseng*	iDPCs and immortalized human outer root sheath cells (ORSCs)	AlamarBlue assay using resazurin (7-hydroxy-3*H*-phenoxazin-3-one 10-oxide) reduction	50 and 100 μg mL^−1^	Iwabuchi et al., 2024 [63]
Extract of leaves of *Gynostemma pentaphyllum*	HFDPCs	CCK-8 assay	50, 100, 200, and 400 μg mL^−1^	Liu et al., 2024 [64]
70% EtOH extract of flowers of *Silybum marianum*	HFDPCs	MTT reduction	50 and 100 μg mL^−1^	You et al., 2024 [65]

* Concentrations at which the plant extract enhanced cell proliferation compared to the vehicle control.

**Table 2:** Effects of plant extracts on the viability of HFDPCs treated with androgens in vitro.

Plant Extracts	Cell Types	Androgens	Assays	Effective Concentrations *	Literature
Extract of *Brassica oleracea*	HFDPCs	50 μg mL^−1^ testosterone	MTT reduction	30 and 100 μg mL^−1^	Luo and Zhang, 2022 [66]
60% EtOH extract of seed cakes of *Camellia japonica*	HFDPCs	10 μg mL^−1^ dihydrotestosterone (DHT)	MTT reduction	10 and 20 μg mL^−1^	Ma et al., 2022 [67]
50% EtOH extract of fruits of *Terminalia bellirica*	HFDPCs	100 μM testosterone	3-(4,5-Dimethyl thiazol-2-yl)-5-(3-carboxymethoxyphenyl)-2- (4-sulpho phenyl)-2H-tetrazolium inner salt (MTS) reduction	6.25, 12.5, and 25 μg mL^−1^	Woo et al., 2023 [68]

* Concentrations at which the plant extract enhanced cell viability compared to the model treated with a hormone.

**Table 3:** Effects of plant extracts on hair follicles ex vivo.

Plant Extracts	Hair Follicles	Hair Growth	Hair Cycle	Cell Proliferation	Literature
Extract of red ginseng (*Panax ginseng*)	Human hair follicles			The extract (100 mg mL^−1^) recovered the number of Ki-67-positive hair matrix keratinocytes reduced by DHT.	Park et al., 2015 [43]
Water extract from oriental melon (*Cucumis melo*) leaves	Human hair follicles	The extract (100 μg mL^−1^) enhanced the elongation of hair (entire hair length).	The extract (100 μg mL^−1^) extended the anagen-phase duration.	The extract (100 μg mL^−1^) increased Ki-67-positive hair bulb keratinocytes.	Pi et al., 2016 [70]
Extract of *Orthosiphon stamineus*	Human hair follicles	The extract (500 μg mL^−1^) enhanced the elongation of hair.	The extract (500 μg mL^−1^) extended the anagen-phase duration.		Somsukskul et al., 2017 [47]
n-Butanol (BuOH) fraction of *Perilla frutescens* extract	C57BL/6 mice vibrissa hair follicles	The BuOH fraction (2.5 μg mL^−1^) enhanced hair shaft growth.			Li et al., 2018 [71]
50% aqueous EtOH extract of *Houttuynia cordata*	Human hair follicles		The extract (20 μg mL^−1^) extended the anagen-phase duration.		Kim et al., 2019 [49]
Extract of *Polygonum multiflorum*	Human hair follicles		The extract (20 or 50 μg mL^−1^) extended the anagen-phase duration.		Shin et al., 2020 [51]
Extract of *Brassica oleracea*	Male C57BL/6 mice hair follicles (whisker pads)	The extract (10 μg mL^−1^) recovered the elongation of the hair shaft suppressed by testosterone.			Luo and Zhang, 2022 [66]
Extract of watercress (*Nasturtium officinale*)	Human hair follicles	The extract (10 mg mL^−1^) enhanced the elongation of hair.			Hashimoto et al., 2022 [72]
Extract of *Panax ginseng*	Human hair follicles	The extract (100 μg mL^−1^) enhanced the elongation of the hair shaft.			Iwabuchi et al., 2024 [63]

**Table 4:** Effects of plant extracts on hair growth in animal models.

Plant Extracts	Animal Models	Vehicle or Formula	Treatments	Hair Growth	Hair Cycle	Literature
Acetone extract of *Boehmeria nipononivea*	5-week-old male C57BL/6 mice; dorsal hair shaving and applying a depilatory agent	EtOH	Topical application; 20 days	Vehicle control < 2% extract		Shimizu et al., 2000 [75]
MeOH extract of dried roots of *Sophora flavescens*	7-week-old female C57BL/6 mice; dorsal hair shaving	50% EtOH	Topical application; 30 days	Vehicle control < 1% extract	↑(telogen to anagen)	Roh et al., 2002 [76]
EtOH extract of roots of *Asiasarum heterotropoides* (or *Asiasarum sieboldi*)	7-week-old female C57BL/6 mice; dorsal hair shaving	40% EtOH	Topical application; 30 days	Vehicle control < 1% extract		Rho et al., 2005 [38]
	7-week-old female C3H mice; dorsal hair shaving	40% EtOH	Topical application; 45 days	Vehicle control < 1% extract		
70% EtOH extract of *Erica multiflora*	7-week-old male C3H/He mice; dorsal hair shaving	Phosphate-buffered saline (PBS)	Subcutaneous injection; 3 weeks	Vehicle control ≤ 0.05% extract	↑(telogen to anagen)	Kawano et al., 2009 [39]
MeOH extract of *Eclipta alba*	62-day-old C57BL/6 mice; dorsal hair shaving	50% propylene glycol (PG), 30% EtOH, and 20% water	Topical application; 10 days	Number of hair follicles; vehicle control < 1.6 mg extract < 3.2 mg extract	↑(telogen to anagen)	Datta et al., 2009 [77]
Extract of tobacco (*Nicotiana tabacum*) leaves microbially biotransformed in cow urine	Male albino Wister rats; dorsal hair shaving and applying a hair remover	Lotion	Topical application; 30 days	Vehicle control ≤ 10% extract ≤ 20% extract ≤ 30% extract ≅ 2% minoxidil		Murkute et al., 2010 [78]
Hot water extract of *Polygonum multiflorum* fermented with *Lactobacillus* sp.	6-week-old C57BL6/N mice; dorsal hair shaving	Water containing *Lactobacillus* sp.	Topical application; 4 weeks	Vehicle control < 4.7 mg extract	↑(telogen to anagen)	Park et al., 2011 [79]
EtOH and aqueous extracts of *Eucheuma cottonii*	10–12-week-old male Sprague–Dawley rats; dorsal hair shaving	Water	Oral administration; 15 days	Vehicle control < aqueous extract < honey < EtOH extract (100 mg kg^−1^)		Fard et al., 2011 [73]
Extract of *Aconiti Ciliare* tubers	7-week-old male C57BL/6 mice; dorsal hair shaving	50% EtOH, 30% water, and 20% PG	Topical application; 35 days	Vehicle control < 2% minoxidil < 1% extract	↑(telogen to anagen)	Park et al., 2012 [40]
Extract of *Glycyrrhiza Glabra*	Female Wistar albino rats; dorsal hair shaving and applying a depilatory cream	Paraffin oil	Topical application; 30 days	Vehicle control < 2% minoxidil < 2% extract	↑(telogen to anagen)	Upadhyay et al., 2012 [80]
Water extract of *Trichosanthes cucumerina* leaves	Wistar albino rats; dorsal hair shaving and applying a depilatory cream	Water	Topical application; 30 days	Vehicle control < 0.03% extract ≤ 2% minoxidil		Sandhya et al., 2012 [81]
Extract of Chinese black tea (*Camellia sinensis* or *Camellia taliensis*) fermented with *Aspergillus* sp.	6-week-old male C3H/He mice; dorsal hair shaving	50% PG, 30% EtOH, and 20% water	Topical application; 2 weeks	Vehicle control ≅ 0.05% capsaicin < 3.5% extract < 0.05% capsaicin plus 3.5% extract		Hou et al., 2013 [82]
Hot water extract of *Thuja orientalis*	6-week-old male C57BL/6N mice; dorsal hair shaving	48.25% PG, 1.75% dimethyl sulfoxide (DMSO), and 50% water	Topical application; 21 days	Vehicle control < 1% minoxidil ≤ 30% extract	↑(telogen to anagen)	Zhang et al., 2013 [83]
Extract from leaves of *Rosmarinus officinalis*	7-week-old male C57BL/6NCrSlc mice; dorsal hair shaving and topical application of testosterone	80% EtOH	Topical application; 30 days	Testosterone model < model with 2% extract ≤ control without testosterone		Murata et al., 2013 [84]
	7-week-old male C3H/He mice; dorsal hair shaving	80% EtOH	Topical application; 30 days	Vehicle control < 2% extract ≤ 1% minoxidil		
Extract of tomato (*Lycopersicon esculentum*)	6-week-old C57BL/6 mice; dorsal hair shaving	10% EtOH	Topical application; 4 weeks	Vehicle control ≅ 3% ethyl acetate extract) < 3% supercritical CO_2_ extract < 3% lycopene-enriched extract ≤ 3% minoxidil	↑(telogen to anagen)	Choi et al., 2013 [85]
Supercritical CO_2_ extract from rice (*Oryza sativa*) brans	6-week-old female C57BL/6 mice; dorsal hair shaving	10% EtOH	Topical application; 4 weeks	Vehicle control < 3% minoxidil ≅ 3% extract	↑(telogen to anagen)	Choi et al., 2014 [86]
EtOH extract from florets of *Carthamus tinctorius*	6-week-old female C57BL/6 mice; dorsal hair shaving	50% PG, 20% EtOH, and 30% water	Topical application; 15 days	Non-treated ≅ vehicle control ≤ 0.05 mg mL^−1^ extract ≤ 0.1 mg mL^−1^ minoxidil ≅ 0.1 mg mL^−1^ extract < 0.5 mg mL^−1^ extract	↑(telogen to anagen)	Junlatat and Sripanidkulchai, 2014 [41]
70% EtOH extract of *Chrysanthemum zawadskii*	8-week-old female C57BL/6 mice; dorsal hair shaving and applying a depilatory cream	70% EtOH	Topical application; 30 days	Vehicle control < 1.6 g kg^−1^ BuOH fraction < 0.6 g kg ^−1^ water fraction	↑(telogen to anagen)	Li et al., 2014 [87]
Extract of *Platycarya strobilacea*	6-week-old male C57BL/6 mice; dorsal hair shaving	DMSO	Topical application; 3 weeks	Vehicle control ≅ 0.1 % extract ≅ 5% minoxidil	↑(telogen to anagen)	Kim et al., 2014 [42]
Extract of *Hibiscus syriacus* leaves	21-day-old albino rats; dorsal hair shaving and applying a depilatory cream	Liquid paraffin	Topical application; 30 days	Vehicle control < 10% extract	↑(telogen to anagen)	Punasiya et al., 2014 [88]
EtOH extract of *Stachytarpheta jamaicensis* leaves	Male Sprague–Dawley rats; dorsal hair shaving	Solution	Topical application; 30 days	Vehicle control < 2% extract ≤ 2% minoxidil		Rozianoor et al., 2014 [89]
Extract of red ginseng (*Panax ginseng*)	7-week-old C57BL/6 mice; dorsal hair shaving	Normal saline	Subcutaneous injection; 7 weeks	Vehicle control < 3% extract ≅ 0.5% minoxidil (topical)	↑(telogen to anagen)	Park et al., 2015 [43]
MeOH extracts of *Chrysanthemum zawadskii* (CZ) and *Polygonum multiflorum* (PM)	7-week-old male athymic BALB/c nude mice	67% PG, 30% EtOH, and 3% DMSO	Topical application; 40 days	Vehicle control ≅ 10 mg PM extract per mouse < 10 mg CZ extract per mouse ≅ 2% minoxidil	↑(telogen to anagen)	Begum et al., 2015 [90]
Hot water extract of herbal mixture: *Acorus calamus*, *Morus alba*, *Glycyrrhiza uralensis*, *Pinus densiflora*, *Sophora angustifolia*, *Ligusticum chuanxiong*, and *Angelica gigas*	7-week-old male C57BL/6 mice; dorsal hair shaving and applying a depilatory cream	Ointment base	Topical application; 18 days	Vehicle control < 5% minoxidil ≤ extract-containing ointment	↑(telogen to anagen)	Park et al., 2015 [91]
Extract of *Rumex japonicus* roots	7-week-old C57BL/6 mice; dorsal hair shaving	60% MeOH and 40% PBS	Topical application; 25 days	Vehicle control ≤ 0.4% extract ≤ 0.8% extract < 5% minoxidil	↑(telogen to anagen)	Lee et al., 2016 [44]
Water extract of oriental melon (*Cucumis melo*) leaves	7-week-old female C57BL/6 mice; dorsal hair shaving	Dulbecco’s phosphate-buffered saline	Topical application; 28 days	Vehicle control < 0.3% extract ≅ 5% minoxidil		Pi et al., 2016 [70]
90% EtOH extract of *Eclipta alba* leaves	Wistar albino rats; dorsal hair shaving and applying a depilatory cream	Water	Topical application; 30 days	Vehicle control < 10% extract ≤ 2% minoxidil		Mondal et al., 2016 [92]
DA-5512 formula (EtOH extract of herbal mixture: *Thea sinensis*, *Emblica officinalis*, *Pinus densiflora*, *Pueraria thunbergiana*, *Tribulus terrestris*, and *Zingiber officinale*)	8-week-old male C57BL/6 mice; dorsal hair shaving and applying a depilatory cream	30% EtOH	Topical application; 14 days	Vehicle control (30% EtOH) < 1% DA-5512 < 3% minoxidil ≅ 5% DA-5512	↑(telogen to anagen)	Yu et al., 2017 [45]
MeOH extract of *Geranium sibiricum*	6-week-old male C57BL/6 mice; dorsal hair shaving	1% DMSO	Topical application; 3 weeks	Vehicle control ≅ 0.1% extract ≅ 5% minoxidil	↑(telogen to anagen)	Boisvert et al., 2017 [46]
Extract of *Trigonella foenum-graecum* leaves	Male albino mice; dorsal hair shaving and applying a depilatory cream	65% water, 25% EtOH, and 10% butylene glycol	Topical application; 21 days	Vehicle control < 5% minoxidil ≤ 10% extract		Imtiaz et al., 2017 [93]
Water extract of *Cinnamomum osmophloeum*	8-week-old male C57BL/6 mice; dorsal hair shaving and applying a calcium thioglycolate solution	Water	Topical spraying; 30 days	Vehicle control < 20% extract ≤ 1% extract ≤ 0.5 mM minoxidil	↑(telogen to anagen)	Wen et al., 2018 [48]
BuOH fraction of *Perilla frutescens* extract	8-week-old C57BL/6 mice; dorsal hair removal by applying a depilatory cream	67% PG, 30% EtOH, and 3% DMSO	Topical application; 25 days	Vehicle control < 2.5% BuOH fraction ≅ 2.5% minoxidil	↑(telogen to anagen)	Li et al., 2018 [71]
	7-week-old male C57BL/6NCrSlc mice; dorsal hair removal and topical application of testosterone or DHT	70% EtOH	Topical application; 15 days	DHT model < testosterone model < DHT with 2 mg BuOH fraction ≤ testosterone with 2 mg BuOH fraction < control without hormones	↑(telogen to anagen)	
Extract of *Serenoa repens*	6–8-week-old male C57BL/6 mice; dorsal hair shaving and applying a depilatory cream	DMSO	Oral administration; 5 weeks	DHT model < model with 50% extract < model with 0.01% finasteride		Zhu et al., 2018 [94]
Extract of blackcurrant (*Ribes nigrum*)	12-week-old ovariectomized female Sprague–Dawley rats	AIN-93M diet	Feeding a diet containing 3% extract; 3 months	Number of hair shafts per follicular unit; ovariectomy control < ovariectomy plus 3% extract ≅ sham control without ovariectomy		Nanashima and Horie, 2019 [95]
60% EtOH extract of *Vernonia anthelmintica* seeds	5–6-week-old male C57BL/6 mice; dorsal hair shaving	0.5% sodium carboxymethylcellulose	Oral administration; 23 days	Chronic restraint stress model < model with 5% minoxidil ≅ model with extract (80 mg kg^−1^)		Wang et al., 2019 [96]
70% EtOH extract of *Camellia sinensis* (CS) leaves and *Hibiscus tilliaceus* (HT) leaves	7–8-week-old male Sprague–Dawley rats; dorsal hair shaving and applying a depilatory cream	Microemulsion	Topical application; 21 days	Vehicle control < 2.5% minoxidil ≤ 7.5% CS extract < 7.5% HT extract		Amin et al., 2019 [97]
EtOH extract of *Angelica gigas*	6–7-week-old male C57/BL6 mice; dorsal hair shaving	Water	Topical application; 17 days	Vehicle control < 0.15% decursin ≅ 2% extract		Lee et al., 2020 [98]
MeOH extract of *Salvia plebeian*	6-week-old male C57BL/6 mice; dorsal hair shaving	DMSO	Topical application; 21 days	Vehicle control < 0.1% extract ≅ 3% minoxidil	↑(telogen to anagen)	Jin et al., 2020 [52]
70% EtOH extract of *Platycladus orientalis* leaves	6-week-old male C57BL/6 mice; dorsal hair shaving	Water	Topical application; 17 days	Vehicle control < 3% extract plus 1% α-terpineol	↑(telogen to anagen)	Ahn et al., 2020 [99]
The extract of *Hibiscus rosa-sinensis*	Sprague–Dawley rats; dorsal hair shaving and applying a depilatory cream	Liquid paraffin	Topical application; 42 days	Vehicle control < 1% extract		Rose et al., 2020 [100]
96% EtOH extract of *Hibiscus rosa-sinensis* leaves	Wistar albino rats; dorsal hair shaving	Liquid paraffin	Topical application; 25 days	Vehicle control < 2.5% extract < 5% extract < 10% extract		Putra et al., 2020 [101]
EtOH extract of *Blumea eriantha*	Male and female Swiss albino mice; dorsal hair shaving	Ag or Fe nanoparticles in 95% EtOH	Topical application; 30 days	Vehicle control < 2% or 5% Fe nanoparticles ≤ 2% or 5% Ag nanoparticles ≤ 2% minoxidil	↑(telogen to anagen)	Chavan et al., 2021 [102]
n-Hexane fraction of the MeOH extract of *Leea indica* leaves	Male and female Swiss albino mice; dorsal hair shaving and applying a surgical hair removal cream	1% Tween 80 in water	Topical application; 21 days	Vehicle control ≤ 5% minoxidil (100 μL) ≤ 1% extract (10 μL)		Sakib et al., 2021 [103]
EtOH and water extracts of *Punica granatum*	Male and female Swiss Albino mice; dorsal hair shaving	95% EtOH	Topical application; 30 days	Vehicle control < 2% minoxidil ≤ 3% extract	↑(telogen to anagen)	Bhinge et al., 2021 [104]
Extract of *Phyllanthus niruri* leaves, *Zingiber officinale* rhizomes, and *Croton tiglium* seeds	6–8-month-old male Wistar rats; dorsal hair shaving	80% EtOH, 10% PG, and 10% water	Topical application; 21 days	Vehicle control < 2% finasteride < 2% extract	↑(telogen to anagen)	Madhunithya et al., 2021 [105]
50% MeOH extract of lotus (*Nelumbo nucifera*) seeds	4-week-old male C57BL/6 mice; dorsal hair shaving	DMSO	Oral administration; 3 weeks	Vehicle control < 3% minoxidil < 0.1% extract	↑(telogen to anagen)	Park et al., 2021 [55]
96% EtOH extract of green tea (*Camellia sinensis*) leaves and celery (*Apium gravelens*) leaves	Guinea pigs; dorsal hair shaving	Tonic	Topical application; 28 days	Vehicle control < hair tonic containing 2.5% green tea extract and 7.5% celery extract		Nursiyah et al., 2021 [106]
Extract of mangkokan (*Nothopanax scutellarium*) leaves	4–5-month-old male New Zealand rabbits; dorsal hair shaving	Lotion	Topical application; 4 weeks	Vehicle control < 2% minoxidil < 10% extract		Rahmi et al., 2021 [107]
Extract of *Pinus thunbergii* barks	7-week-old male C57BL/6 mice; dorsal hair shaving	Water	Topical application; 17 days	Vehicle control < 1% minoxidil (100 μL) < 2% extract ≅ 4% extract		Her et al., 2022 [108]
Extract of centipedegrass (*Eremochloa ophiuroides*)	6-week-old female C57BL/6 mice; dorsal hair shaving	50% glycerol, 25% EtOH, and 25% water	Topical application; 14 days	Vehicle control < 1% extract < 5% minoxidil	↑(telogen to anagen)	Ramadhani et al., 2022 [56]
EtOH extract of *Blumea eriantha*	Male and female albino mice; dorsal hair shaving	95% EtOH	Topical application; 30 days	Control (normal saline) < 1% extract ≤ 1% minoxidil ≤ 3% extract	↑(telogen to anagen)	Bhinge et al., 2022 [109]
60% EtOH extract of camellia (*Camellia japonica*) seed cakes	7-week-old male C57BL/6J mice; dorsal shaving and applying 6% Na_2_S solution	Water	Topical application; 21 days	Vehicle control < 10% extract < 5% minoxidil		Wang et al., 2022 [58]
Hot water extract of *Lycopus lucidus*	Female 7-week-old male C57BL/6 mice; dorsal hair shaving	Diet	Oral feeding; 5 weeks	Control diet < diet supplemented with 0.01% extract		Lee et al., 2022 [59]
Hot water extract of mango (*Mangifera Indica*) leaves	8-week-old male C57BL/6J mice; dorsal hair shaving and applying a depilatory cream	82.5% water, 12.5% EtOH, and 0.05% jojoba oil	Topical application; 11 days	Vehicle control < 1% extract ≤ 0.3% minoxidil		Jung et al., 2022 [110]
96% EtOH extract of terentang (*Campnosperma brevipetiolatum*) stem barks	Male rabbits; dorsal hair shaving	Water	Topical application; 21 days	Vehicle control < 0.5% extract < 1% extract < 5% extract ≤ 5% minoxidil		Gunawan et al., 2022 [111]
EtOH extract of sea hibiscus (*Hibiscus tileaceus*) leaves	Male guinea pigs; dorsal hair shaving	Tonic	Topical application; 3 weeks	Vehicle control < 30% extract ≤ 2% minoxidil		Leny et al., 2022 [112]
Cold vacuum extract of *Notocactus ottonis*	8-week-old male C57BL/6 mice; dorsal hair shaving	50% PG, 30% EtOH, and 20% water	Topical application; 27 days	Vehicle control < 10% extract ≤ 5% minoxidil		Shibato et al., 2023 [113]
EtOH extract of *Terminalia bellirica* fruits	7-week-old male C57BL/6 mice; dorsal hair shaving, applying a depilatory cream, and subcutaneous injection of testosterone	Water	Oral administration; 14 days	Testosterone model ≅ model with 2 mg kg^−1^ finasteride < model with 20 mg kg^−1^ extract ≅ model with 100 mg kg^−1^ extract < control without testosterone		Woo et al., 2023 [68]
50% EtOH extract of *Cudrania tricuspidata* and *Sargassum fusiforme*	7-week-old female C57BL/6 mice; dorsal hair shaving and applying a depilatory cream	Water	Oral administration; 21 days	Vehicle control < 50 mg kg^−1^ extract < 60 μg kg^−1^ minoxidil		Rajan et al., 2023 [74]
			Topical application; 21 days	Vehicle control < 250 mg kg^−1^ minoxidil < 50 mg kg^−1^ extract		
75% EtOH extract of *Carica papaya* leaves	Sprague–Dawley rats	Ointment base	Topical application; 30 days	Vehicle control < 2% minoxidil < 5% extract		Dangi et al., 2023 [114]
96% EtOH extract of *Capsicum frutescens* leaves	Male rabbits; dorsal hair shaving and applying a depilatory cream	Tonic	Topical application; 21 days	Vehicle control < 20% extract ≤ 2% minoxidil		Tendri Adjeng et al., 2023 [115]
70% EtOH extract of *Hibiscus rosa-sinensis* leaves	White rabbits; dorsal hair shaving	Cream	Topical application; 21 days	Vehicle control < 20% extract ≤ a minoxidil product		Lailiyah, 2023 [116]
Extract of *Gynostemma pentaphyllum* leaves	4-week-old male C57BL/6 mice; dorsal hair shaving	Water	Topical application; 28 days	Vehicle control ≅ 0.5% extract ≤ 2% minoxidil < 1% extract ≅ 2% extract	↑(telogen to anagen)	Liu et al., 2024 [64]

<, ≤, and ≅ represent big differences, little differences, and no difference, respectively. ↑ represents increases.

**Table 5:** Effects of plant extracts on hair growth in clinical trials.

Study Format and Subjects	Plant Extracts	Formulas	Treatments	Outcomes	Literature
Double-blind, randomized, placebo-controlled trial on 44 subjects with male or female pattern alopecia (aged 18 to 60 years)	Extract from *Thuja occidentalis* seeds	A shampoo containing 0.17% extract	Topical application; twice daily for 16 weeks	The shampoo increased total hair count compared to the placebo group.	Baek et al., 2011 [118]
Double-blind, randomized, placebo-controlled trial on 50 women subjects (aged 18 years or over)	Extract from barks of *Stryphnodendron adstringens*	A cream containing 6.0% extract	Topical application; twice daily for 6 months	The cream reduced terminal hair growth.	Vicente et al., 2009 [120]
Double-blind, randomized, controlled single-center trial on 50 alopecia patients including 22 women and 28 men (aged 18 years or over, 42.0 ± 11.37 years)	Supercritical CO_2_ extract of brans of *Oryza sativa*	A tonic containing 0.5% extract	Topical application; twice a day for 16 weeks	The tonic increased hair diameter and the density of hairs per skin area in male subjects.	Choi et al., 2015 [121]
Double-blind, placebo-controlled, randomized clinical trial on 23 subjects with mild alopecia (aged 20 to 60 years)	DA-5512 formula (EtOH extract of herbal mixture: *Thea sinensis*, *Emblica officinalis*, *Pinus densiflora*, *Pueraria thunbergiana*, *Tribulus terrestris*, and *Zingiber officinale*)	A solution	Topical application on the shaved head skin twice daily for 16 weeks	Hair density, hair shaft diameter, and hair growth rate; placebo (n = 8) < 5% DA-5512 (n = 8) ≅ 3% minoxidil (n = 7).	Yu et al., 2017 [45]
Double-blind, randomized, placebo-controlled study on 30 women (aged 20 to 52 years)	n-Hexane extract of *Curcuma aeruginosa*	A lotion containing 5% extract	Topical application; twice daily for 12 weeks	The lotion reduced the growth rates of axillary hairs.	Srivilai et al., 2018 [122]
Randomized, placebo-controlled, single-blind, clinical study on 120 subjects with androgenetic alopecia and telogen effluvium (aged 20 to 55 years, 36.9 ± 9.8 years)	A mixture of herbal extracts: *Urtica urens*, *Urtica dioica*, *Matricaria chamomilla*, *Achillea millefolium*, *Ceratonia siliqua*, and *Equisetum arvense.*	A shampoo and a solution	Topical application of active shampoo (3 to 4 min), 3 times a week, and/or active solution (4 to 6 h) daily for 6 months	Effectiveness in preventing and reducing hair loss; placebo shampoo plus placebo solution (n = 30) < active shampoo (n = 30) ≤ active solution (n = 30) ≤ active shampoo plus active solution (n = 30).	Pekmezci et al., 2018 [123]
Double-blind, randomized controlled study on 47 subjects including male and female patients with androgenic alopecia (aged 18 to 54 years)	Extracts of *Inula helenium* (IH) roots and *Caesalpinia sappan* (CS) barks	A shampoo containing 0.3% IH root extract and 0.1% CS bark extract	Topical application twice daily for 24 weeks	The treatment group (n = 23) showed a higher hair density and total hair count than the placebo group (n = 24).	Choi et al., 2019 [124]
Randomized, double-blind, placebo-controlled study on 72 patients with mild to moderate vertex balding (aged 37 to 54 years, 46.6 ± 8.5 years)	Extract of *Centipeda minima*	A tonic	Topical application daily for 24 weeks	The treatment group (n = 34) showed a higher hair count than the placebo group (n= 32).	Kim et al., 2020 [125]
Double-blind, randomized controlled study on 46 male subjects (aged 20 to 55 years)	Extract of watercress (*Nasturtium officinale*)	A lotion containing 2% extract	Topical application twice daily for 6 months	The treatment group (n = 23) showed a higher hair thickness and hair density than the placebo group (n = 23).	Hashimoto et al., 2022 [72]
Randomized, double-blind, placebo-controlled clinical study on 50 subjects including 7 males and 43 females (aged 20 years or over)	Water extract of banana (*Musa* *paradisiaca*) flowers	A sachet containing 16% extract	Oral administration daily for 12 weeks	The sachet uptake increased the hair root diameter and reduced hair loss and scalp redness compared to the placebo group.	Liang et al., 2023 [62]
Randomized, double-blind, placebo-controlled clinical study on 88 subjects including 34 males and 54 females (aged 19 to 60 years, 38.52 ± 7.98 years)	Extract of persimmon (*Diospyros kaki*) leaves, green tea (*Camellia sinensis*) leaves, and sophora (*Sophora Japonica*) fruits	A tablet containing 30% extract	Oral administration twice daily for 24 weeks	The treatment group (n = 44) showed a higher hair density and hair diameter compared with the placebo group (n = 44).	Ham et al., 2023 [126]
Randomized, double-blind, placebo-controlled clinical study on 42 subjects including male and female patients with androgenetic alopecia (aged 18 to 54 years, 46.096 ± 6.60 years)	EtOH extract from flowers of *Silybum marianum*	A shampoo containing 0.05% extract	Topical application; once a day for 24 weeks	The shampoo increased the hair density and total hair count compared with those in the placebo group.	You et al., 2024 [65]

<, ≤, and ≅ represent big differences, little differences, and no difference, respectively.

**Table 6:** Main phytochemical components and active compounds in plant extracts.

Plant Extracts	Main Phytochemical Components and Active Compounds	Literature
Acetone extract of *Boehmeria nipononivea*	**α-Linolenic acid**, linoleic acid, palmitic acid, **elaidic acid**, oleic acid, and **stearic acid**	Shimizu et al., 2000 [75]
MeOH extract of *Eclipta alba*	Coumestans (e.g., **Wedelolactone**), flavonoids, triterpenoid glycosides, triterpenoid saponins, and thiophene derivatives	Datta et al., 2009 [77]
Hot water extract of *Thuja orientalis*	Kaempferol and isoquercetin	Zhang et al., 2013 [83]
Extract of *Rosmarinus officinalis* leaves	**12-Methoxycarnosic acid** (a diterpenoid)	Murata et al., 2013 [84]
Extract of tomato (*Lycopersicon esculentum*)	**all-*trans*-Lycopene** and **5-*cis-*lycopene**	Choi et al., 2013 [85]
Supercritical CO_2_ extract of rice (*Oryza sativa*) brans	**Linoleic acid**, policosanol, γ-oryzanol, and γ-tocotrienol	Choi et al., 2014 [86]
50% EtOH extract of *Carthamus tinctorius* florets	Hydroxysafflor yellow A (a chalcone glycoside)	Junlatat and Sripanidkulchai, 2014 [41]
EtOH extract of *Stachytarpheta jamaicensis* leaves	Genipin, phytol, α-linolenic acid, palmitic acid, and tridecanoic acid	Rozianoor et al., 2014 [89]
Extract of red ginseng (*Panax ginseng*)	**Ginsenoside Rb1** and **ginsenoside Rg3**	Park et al., 2015 [43]
Hot water extract of an herbal mixture: *Acorus calamus*, *Morus alba*, *Glycyrrhiza uralensis*, *Pinus densiflora*, *Sophora angustifolia*, *Ligusticum chuanxiong*, and *Angelica giga*	Asarone and *p-*coumaric acid	Park et al., 2015 [91]
MeOH extract of *Geranium sibiricum*	Corilagin and gallic acid	Boisvert et al., 2017 [46]
Water extract of *Cinnamomum osmophloeum*	Cinnamic aldehyde and cinnamic acid	Wen et al., 2018 [48]
BuOH fraction of *Perilla frutescens* extract	**Rosmarinic acid**	Li et al., 2018 [71]
n-Hexane extract of *Curcuma aeruginosa*	**Germacrone** and other sesquiterpenoids (e.g., dehydrocurdione, zederone, cucumenone, curcumenol, and furanodiene)	Srivilai et al., 2018 [122]
A mixture of herbal extracts: *Urtica urens*, *Urtica dioica*, *Matricaria chamomilla*, *Achillea millefolium*, *Ceratonia siliqua*, and *Equisetum arvense*	Kaempferol, quercetin, and myricetin	Pekmezci et al., 2018 [123]
Extracts of *Inula helenium* (IH) roots and *Caesalpinia sappan* (CS) barks	Costunolide (from IH) and 3-deoxysappanchalcone (from CS)	Choi et al., 2019 [124]
Extract of Centipeda minima	**Brevilin A** and other sesquiterpene lactones (e.g., arnicolide C, arnicolide D, and microhelenin C)	Kim et al., 2020 [125]
EtOH extract of *Angelica gigas*	**Decursin** and decursinol angelate	Lee et al., 2020 [98]
n-Hexane fraction of the MeOH extract of *Leea indica leaves*	**Phthalic acid**, palmitic acid, n-octadecane, n-eicosane, n-heptadecane, and farnesol	Sakib et al., 2021 [103]
EtOH and water extract of *Punica granatum*	Volatile compounds (e.g., maltol and 5-hydroxymethylfurfural)	Bhinge et al., 2021 [104]
MeOH extract of shallot (*Allium ascalonicum*)	**Rosmarinic acid**, *p-*coumaric acid, and quercetin	Ruksiriwanich et al., 2022 [57]
Extract of *Brassica oleracea*	**Sulforaphane** and **glucoraphanin** (a glucosinolate of sulforaphane)	Luo and Zhang, 2022 [66]
EtOH extract of *Blumea eriantha*	**Dimethyl sulfone**	Bhinge et al., 2022 [109]
60% EtOH extract of seed cakes of *Camellia japonica*	Kaempferol-3-*O-*[2-*O-*β-D-galactopyranosyl-6-*O-*α-L-rhamnopyranosyl]-β-D-glucopyranoside and kaempferol-3-*O*-[2-*O*-β-D-xylopyranosyl-6-*O*-α-L-rhamnopyranosyl]-β-D-glucopyranoside	Ma et al., 2022 [67]
Hot water extract of *Lycopus lucidus*	**Rosmarinic acid**	Lee et al., 2022 [59]
70% EtOH extract of fruit shells of *Camellia japonica*	Protocatechuic acid gallic acid	You et al., 2023 [61]
Extract of persimmon (*Diospyros kaki*) leaves, green tea (*Camellia sinensis*) leaves, and sophora (*Sophora Japonica*) fruits	Tannic acids (from persimmon), (−)-epigallocatechin-3-gallate (from green tea), and sophoricoside (from sophora)	Ham et al., 2023 [126]
Extract of *Panax ginseng*	**Ginsenoside Rb1**, **ginsenoside Rg1**, and **ginsenoside Re**	Iwabuchi et al., 2024 [63]
70% EtOH extract of flowers of *Silybum marianum*	Apigenin	You et al., 2024 [65]

Active compounds with experimental evidence are indicated with bold letters.

**Table 7:** Antioxidant, anti-inflammatory, and anti-senescence effects of plant extracts.

Plant Extract Sources	Model	Antioxidant, Anti-Inflammatory, and Anti-Senescence Effects	Literature
*Platycarya strobilacea*	In vitro	The extract had 1,1-diphenyl-2-picrylhydrazyl (DPPH) radical-scavenging capacity.	Kim et al., 2014 [42]
*Geranium sibiricum*	In vitro	The extract had a DPPH radical-scavenging capacity.	Boisvert et al., 2017 [46]
*Panax ginseng*, *Glycine max*, *Houttuynia cordata*, *Lycium chinense*, *Glycyrrhiza uralensis*, *Citrus unshiu*, *Zizyphus jujuba*, *Perilla frutescens*, *Camellia sinensis*, and *Cynanchum wilfordii*	Mast cell-1	The extract suppressed the production of tumor necrosis factor-alpha (TNF-α) in cells stimulated with phorbol-12-myristate 13-acetate (PMA) plus calcium ionophore A23187.	Kang et al., 2019 [50]
*Angelica gigas*	Male C57/BL6 mice	The extract reduced pro-inflammatory cytokines, such as TNF-α and interleukin (IL)-1β, while increasing anti-inflammatory cytokines, such as IL-4 and IL-13, in the dorsal skin.	Lee et al., 2020 [98]
*Leea indica*	In silico	Molecular docking analysis identified some phytochemicals, such as including phthalic acid, that showed high ligand efficiencies towards prostaglandin D_2_ synthase.	Sakib et al., 2021 [103]
*Euterpe oleracea*, *Olea europea*, *Tabebuia impetiginosa*, and *Coffea Arabica*	HFDPCs	The extract enhanced the viability of cells exposed to 2,2’-azobis (2-amidinopropane) dihydrochloride (AAPH) radical.	Serruya and Maor, 2021 [54]
*Nelumbo nucifera*	In vitro	The extract had a DPPH radical-scavenging capacity.	Park et al., 2021 [55]
*Pinus thunbergii*	Male C57/BL6 mice	The extract reduced pro-inflammatory cytokines, such as TNF-α and IL-1β, while increasing anti-inflammatory cytokines, such as IL-4 and IL-13, in the dorsal skin.	Her et al., 2022 [108]
*Camellia japonica*	HFDPCs	The extract suppressed the production of IL-6 and IL-1α in cells stimulated with DHT. It also reduced the expression of senescence-associated β-galactosidase (SA-β-gal) in DHT-treated cells.	Ma et al., 2022 [67]
*Lycopus lucidus*	HFDPCs	The extract reduced IL-1β levels in cells exposed to hydrogen peroxide (H_2_O_2_).	Lee et al., 2022 [59]
*Camellia japonica*	In vitro	The extract had DPPH radical-scavenging capacity.	You et al., 2023 [61]
	HFDPCs	The extract reduced intracellular reactive oxygen species (ROS) levels and enhanced the viability of cells exposed to H_2_O_2_. It reduced SA-β-gal expression in cells exposed to H_2_O_2_.	
*Musa paradisiaca*	HFDPCs	The extract reduced intracellular ROS levels exposed to H_2_O_2_.	Liang et al., 2023 [62]
*Coffea arabica*	In vitro	The extract had 2,2’-azino-bis(3-ethylbenzothiazoline-6-sulfonic acid (ABTS) radical and DPPH radical-scavenging capacities.	Muangsanguan et al., 2023 [135]
*Silybum marianum*	HFDPCs	The extract reduced intracellular ROS levels and enhanced the viability of cells exposed to H_2_O_2_. It reduced the expression of SA-β-gal and IL-6 in senescent cells and young cells exposed to H_2_O_2_.	You et al., 2024 [65]

**Table 8:** Effects of plant extracts on apoptosis pathway.

Plant Extract Sources	Models	BCL-2	BAX	BAD	Literature
*Rumex japonicus*	HFDPCs	↑(protein)	↓(protein)		Lee et al., 2016 [44]
*Serenoa repens*	C57BL/6 mice	↑(protein)	↓(protein)		Zhu et al., 2018 [94]
*Houttuynia cordata*	HFDPCs	↑(mRNA) ↑(protein)	↓(mRNA)	=(mRNA)	Kim et al., 2019 [49]
*Panax ginseng*, *Glycine max*, *Houttuynia cordata*, *Lycium chinense*, *Glycyrrhiza uralensis*, *Citrus unshiu*, *Zizyphus jujuba*, *Perilla frutescens*, *Camellia sinensis*, and *Cynanchum wilfordii*	HFDPCs		↓(mRNA) ↓(protein)		Kang et al., 2019 [50]
*Polygonum multiflorum*	HFDPCs	↑(mRNA)		↓(mRNA)	Shin et al., 2020 [51]
*Salvia plebeia*	HFDPCs	↑(protein)	=(protein)		Jin et al., 2020 [52]
*Brassica oleracea*	HFDPCs	=(mRNA)	↓(mRNA)		Luo and Zhang, 2022 [66]
*Camellia japonica*	HFDPCs	↑(mRNA) ↑(protein)	↓(mRNA)		Ma et al., 2022 [67]

↑, ↓, and = represent increases, decreases, and no changes, respectively. Abbreviations: BCL-2—B-cell lymphoma 2; BAX—BCL-2-associated X protein; BAD—BCL-2-associated agonist of cell death.

**Table 9:** Effects of plant extracts on androgens, their receptors, and steroid 5α-reductase type II.

Plant Extract Sources	Models	Testosterone	Androgen Receptor	Steroid 5α-Reductase Type II	Literature
*Sophora flavescens*	In vitro			↓(activity)	Roh et al., 2002 [76]
*Nicotiana tabacum*	Male albino Wister rats	↓(protein)			Murkute et al., 2010 [78]
*Rosmarinus officinalis*	In vitro			↓(activity)	Murata et al., 2013 [84]
*Panax ginseng*	HFDPCs		↓(mRNA)		Park et al., 2015 [43]
*Panax ginseng*, *Glycine max*, *Houttuynia cordata*, *Lycium chinense*, *Glycyrrhiza uralensis*, *Citrus unshiu*, *Zizyphus jujuba*, *Perilla frutescens*, *Camellia sinensis*, and *Cynanchum wilfordii*	HFDPCs			↓(protein)	Kang et al., 2019 [50]
*Polygonum multiflorum*	HFDPCs		↓(protein)		Shin et al., 2020 [51]
*Plumbago zeylanica*	HFDPCs			↓(protein)	Yamada et al., 2020 [53]
*Allium ascalonicum*	Prostate cancer cell line Du-145			↓(mRNA)	Ruksiriwanich et al., 2022 [57]
*Mangifera indica*	HFDPCs			↓(mRNA)	Jung et al., 2022 [110]
*Camellia japonica*	HFDPCs		↓(mRNA)	↓(mRNA)	Ma et al., 2022 [67]
	In vitro			↓(activity)	You et al., 2023 [61]
*Musa paradisiaca*	HFDPCs		↓(mRNA)	↓(mRNA)	Liang et al., 2023 [62]
*Coffea arabica*	HFDPCs			↓(mRNA)	Muangsanguan et al., 2023 [135]

↓ represents decreases.

**Table 10:** Effects of plant extracts on cell cycle.

Plant Extract Sources	Models	CDKs	p16 ^INK4^	p53	Cell Cycle Phase	Literature
*Erica multiflora*	HFDPCs				↓(G0/G1), =(S), ↑(G2/M)	Kawano et al., 2009 [39]
*Houttuynia cordata*	HFDPCs	=(mRNA, CDK1 and CDK2), ↑(mRNA, CDK4)	↓(protein)	=(mRNA)		Kim et al., 2019 [49]
*Camellia japonica*	HFDPCs			↓(mRNA)		Ma et al., 2022 [67]
	HFDPCs				↓(G0/G1), ↑(S), ↑(G2/M)	Wang et al., 2022 [58]

↑, ↓, and = represent increases, decreases, and no changes, respectively. Abbreviations: CDK—cyclin-dependent kinase; INK—inhibitors of CDK.

**Table 11:** Effects of plant extracts on the mRNA and protein levels of several growth factors.

Plant Extract Sources	Models	IGF-1	VEGF	HGF	KGF (FGF-7)	Literature
*Sophora flavescens*	HFDPCs	↑(mRNA)	=(mRNA)	=(mRNA)	↑(mRNA)	Roh et al., 2002 [76]
*Asiasarum heterotropoides*	HFDPCs	=(mRNA)	↑(mRNA)	=(mRNA)	=(mRNA)	Rho et al., 2005 [38]
*Eclipta alba*	C57BL/6 mice				↑(protein)	Datta et al., 2009 [77]
*Lycopersicon esculentum*	C57BL/6 mice	↑(mRNA)	↑(mRNA)		↑(mRNA)	Choi et al., 2013 [85]
*Oryza sativa*	C57BL/6 mice	↑(mRNA)	↑(mRNA)		↑(mRNA)	Choi et al., 2014 [86]
*Carthamus tinctorius*	HFDPCs		↑(mRNA)		↑(mRNA)	Junlatat and Sripanidkulchai, 2014 [41]
*Platycarya strobilacea*	HFDPCs	↓(mRNA)			=(mRNA)	Kim et al., 2014 [42]
*Panax ginseng*	HFDPCs	=(mRNA)	=(mRNA)	=(mRNA)		Park et al., 2015 [43]
*Acorus calamus*, *Morus alba*, *Glycyrrhiza uralensis*, *Pinus densiflora*, *Sophora angustifolia*, *Ligusticum chuanxiong*, and *Angelica gigas*	C57BL/6 mice		↑(mRNA)		↑(mRNA)	Park et al., 2015 [91]
*Geranium sibiricum*	HFDPCs		↑(mRNA)	↑(mRNA)		Boisvert et al., 2017 [46]
	C57BL/6 mice		↓(mRNA)	↓(mRNA)		
*Biota orientalis*, *Eclipta thermalis*, *Sophora angustifolia*, *Cnidium monnieri*, *Ligusticum chuanxiong*, and *Panax notoginseng*	HFDPCs		↑(mRNA)		↑(mRNA)	Zeng et al., 2017 [148]
*Cinnamomum osmophloeum*	HFDPCs	=(mRNA)	↑(mRNA)	=(mRNA)	↑(mRNA)	Wen et al., 2018 [48]
*Houttuynia cordata*	HFDPCs	=(protein)	↑(protein)		=(protein)	Kim et al., 2019 [49]
*Polygonum multiflorum*	HFDPCs		↑(protein)			Shin et al., 2020 [51]
*Salvia plebeia*	HFDPCs			↑(mRNA)		Jin et al., 2020 [52]
*Platycladus orientalis*	C57BL/6 mice	↑(protein)	↑(protein)			Ahn et al., 2020 [99]
*Nelumbo nucifera*	C57BL/6 mice	↑(mRNA)	↑(mRNA)			Park et al., 2021 [55]
*Centipeda minima*	HFDPCs	↑(protein)	↑(mRNA) ↑(protein)			Kim et al., 2021 [149]
*Brassica oleracea*	HFDPCs	=(mRNA)	↑(mRNA)		↓(mRNA)	Luo and Zhang, 2022 [66]
*Pinus thunbergii*	C57BL/6 mice	↑(protein)	↑(protein)			Her et al., 2022 [108]
*Eremochloa ophiuroides*	HFDPCs	↑(mRNA)	↑(mRNA)			Ramadhani et al., 2022 [56]
*Allium ascalonicum*	HFDPCs		↑(mRNA)			Ruksiriwanich et al., 2022 [57]
*Camellia japonica*	HFDPCs	↑(mRNA)	↑(mRNA)	↑(mRNA)		Wang et al., 2022 [58]
*Lycopus lucidus*	HFDPCs		↑(protein) =(mRNA)			Lee et al., 2022 [59]
	C57BL/6 mice	↑(protein)	↑(protein)			
*Camellia japonica*	HFDPCs		↑(mRNA) ↓(protein)			You et al., 2023 [61]
*Coffea arabica*	HFDPCs		↑(mRNA)			Muangsanguan et al., 2023 [135]
*Cudrania tricuspidata* and *Sargassum fusiforme*	C57BL/6 mice		↑(mRNA)			Rajan et al., 2023 [74]
*Silybum marianum*	HFDPCs	↑(mRNA)	↑(protein)		↑(mRNA)	You et al., 2024 [65]

↑, ↓, and = represent increases, decreases, and no changes, respectively. Abbreviations: IGF—insulin-like growth factor; VEGF—vascular endothelial growth factor; HGF—hepatocyte growth factor; KGF—keratinocyte growth factor; FGF-7—fibroblast growth factor 7.

**Table 12:** Effects of plant extracts on the AKT and mitogen-activated protein kinase (MAPK) signaling pathways.

Plant Extract Sources	Models	AKT	ERK	JNK	p38 MAPK	Literature
*Panax ginseng*	HFDPCs	↑(phospho)	↑(phospho)			Park et al., 2015 [43]
*Rumex japonicus*	HFDPCs	↑(phospho)	↑(phospho)	=(phospho)	=(phospho)	Lee et al., 2016 [44]
*Houttuynia cordata*	HFDPCs	↑(phospho)	↑(phospho)			Kim et al., 2019 [49]
*Salvia plebeia*	HFDPCs	↑(phospho)	↑(phospho)			Jin et al., 2020 [52]
*Euterpe oleracea*, *Olea europea*, *Tabebuia impetiginosa*, and *Coffea Arabica*	HFDPCs		↑(phospho)			Serruya and Maor, 2021 [54]
*Eremochloa ophiuroides*	HFDPCs	↑(phospho)				Ramadhani et al., 2022 [56]
*Camellia japonica*	HFDPCs	↑(phospho)	↑(phospho)			Wang et al., 2022 [58]
*Centipeda minima*	HFDPCs		↑(phospho)	↑(phospho)	↓(phospho)	Kim et al., 2021 [149]

↑, ↓, and = represent increases, decreases, and no changes, respectively. Abbreviations: AKT—protein kinase B (PKB); ERK—extracellular signal-regulated kinases; JNK—c-Jun N-terminal kinase; p38 MAPK—p38 mitogen-activated protein kinase; phospho—phosphorylation.

**Table 13:** Effects of plant extracts on the mediators of the WNT signaling pathways.

Plant Extract Sources	Models	WNTs	DKK1	GSK3β	β-Catenin	LEF1	c-Myc	Cyclin D1	Literature
*Polygonum multiflorum*	C57BL6/N				↑(protein)				Park et al., 2011 [79]
*Aconiti Ciliare*	iDPCs				↑(protein)				Park et al., 2012 [40]
*Thuja orientalis*	C57BL/6N mice				↑(protein)				Zhang et al., 2013 [83]
*Rumex japonicus*	HFDPCs			↑(phospho)	↑(protein)				Lee et al., 2016 [44]
*Polygonum multiflorum*	HFDPCs		↓(protein)						Shin et al., 2020 [51]
*Salvia plebeia*	HFDPCs			↑(phospho)	↓(phospho) ↑(protein)				Jin et al., 2020 [52]
*Platycladus orientalis*	C57BL/6 mice	WNT3 ↑(protein)			↑(protein)				Ahn et al., 2020 [99]
*Centipeda minima*	HFDPCs	WNT5a ↑(mRNA)		↑(phospho)	↑(protein)				Kim et al., 2021 [149]
*Brassica oleracea*	HFDPCs				=(mRNA)				Luo and Zhang, 2022 [66]
*Eremochloa ophiuroides*	HFDPCs			↑(phospho)	↑(protein)				Ramadhani et al., 2022 [56]
*Allium ascalonicum*	HFDPCs				↑(mRNA)				Ruksiriwanich et al., 2022 [57]
*Mangifera indica*	HFDPCs		↓(mRNA)				↑(mRNA)		Jung et al., 2022 [110]
*Nasturtium officinale*	Human hair follicles		↓(protein)						Hashimoto et al., 2022 [72]
*Camellia japonica*	HFDPCs	WNT1 ↑(mRNA)	↓(protein)				↑(mRNA)	↑(mRNA)	You et al., 2023 [61]
*Terminalia bellirica*	C57BL/6 mice				↑(protein)			↑(protein)	Woo et al., 2023 [68]
*Cudrania tricuspidata* and *Sargassum fusiforme*	C57BL/6 mice	WNT5a, WNT7b ↑(mRNA)							Rajan et al., 2023 [74]
*Gynostemma pentaphyllum*	HFDPCs	WNT5a ↑(mRNA) ↑(protein)	↓(mRNA)		↑(mRNA) ↑(protein)	↑(mRNA)			Liu et al., 2024 [64]

↑, ↓, and = represent increases, decreases, and no changes, respectively. Abbreviations: WNT—Wingless and Int-1; DKK1—dickkopf 1; GSK3β—glycogen synthase kinase 3β; LEF1—lymphoid enhancer-binding factor 1.

**Table 14:** Effects of plant extracts on the mediators of the sonic hedgehog (SHH) signaling pathways.

Plant Extract Sources	Models	SHH	SMO	GLI1	Literature
*Eclipta alba*	C57BL/6 mice	↑(protein)			Datta et al., 2009 [77]
*Polygonum multiflorum*	C57BL6/N	↑(protein)			Park et al., 2011 [79]
*Thuja orientalis*	C57BL/6N mice	↑(protein)			Zhang et al., 2013 [83]
*Eremochloa ophiuroides*	C57BL/6 mice	↑(protein)			Ramadhani et al., 2022 [56]
*Allium ascalonicum*	HFDPCs	↑(mRNA)	↑(mRNA)	↑(mRNA)	Ruksiriwanich et al., 2022 [57]
*Coffea arabica*	HFDPCs	↑(mRNA)	↑(mRNA)	↑(mRNA)	Muangsanguan et al., 2023 [135]

↑ represents increases. Abbreviations: SMO—smoothened; GLI—glioma-associated oncogene transcription factor.

**Table 15:** Effects of plant extracts on the TGF-β and BMP signaling pathways.

Plant Extract Sources	Models	TGF-β1	TGF-β2	BMP4	SMAD2	SMAD3	Literature
*Asiasarum heterotropoides*	HFDPCs	=(mRNA)					Rho et al., 2005 [38]
*Eclipta alba*	C57BL/6 mice			↓(protein)			Datta et al., 2009 [77]
*Lycopersicon esculentum*	C57BL/6 mice	=(mRNA)					Choi et al., 2013 [85]
*Oryza sativa*	C57BL/6 mice	↓(mRNA)					Choi et al., 2014 [86]
*Carthamus tinctorius*	HFDPCs	↓(mRNA)					Junlatat and Sripanidkulchai, 2014 [41]
*Platycarya strobilacea*	HFDPCs	=(mRNA)					Kim et al., 2014 [42]
*Geranium sibiricum*	HFDPCs	=(mRNA)					Boisvert et al., 2017 [46]
	C57BL/6 mice	↓(mRNA)					
*Cinnamomum osmophloeum*	HFDPCs		↑(mRNA)				Wen et al., 2018 [48]
*Serenoa repens*	C57BL/6 mice		↓(protein)				Zhu et al., 2018 [94]
*Panax ginseng*, *Glycine max*, *Houttuynia cordata*, *Lycium chinense*, *Glycyrrhiza uralensis*, *Citrus unshiu*, *Zizyphus jujuba*, *Perilla frutescens*, *Camellia sinensis*, and *Cynanchum wilfordii*	HFDPCs	↓(mRNA)					Kang et al., 2019 [50]
*Salvia plebeia*	HFDPCs	↓(mRNA)			↓(protein)	↓(protein)	Jin et al., 2020 [52]
*Euterpe oleracea*, *Olea europea*, *Tabebuia impetiginosa*, and *Coffea Arabica*	HFDPCs	↓(protein)					Serruya and Maor, 2021 [54]
*Nelumbo nucifera*	C57BL/6 mice	↓(mRNA)					Park et al., 2021 [55]
*Brassica oleracea*	HFDPCs	=(mRNA)					Luo and Zhang, 2022 [66]
*Camellia japonica*	HFDPCs	↓(mRNA)					Wang et al., 2022 [58]
*Acorus calamus*, *Morus alba*, *Glycyrrhiza uralensis*, *Pinus densiflora*, *Sophora angustifolia*, *Ligusticum chuanxiong*, and *Angelica gigas*	C57BL/6 mice	↓(mRNA)					Muangsanguan et al., 2023 [135]
*Panax ginseng*				↓(mRNA)			Iwabuchi et al., 2024 [63]
*Gynostemma pentaphyllum*	HFDPCs	↓(mRNA) ↓(protein)					Liu et al., 2024 [64]
*Silybum marianum*	HFDPCs	↓(mRNA)					You et al., 2024 [65]

↑, ↓, and = represent increases, decreases, and no changes, respectively. Abbreviations: TGF—transforming growth factor; BMP—bone morphogenetic factor.

## Data Availability

The original contributions presented in this study are included in the article, and further inquiries can be directed to the corresponding author.

## References

[1] Yang F.C., Zhang Y., Rheinstädter M.C. (2014). The structure of people’s hair. PeerJ.

[2] Lin X.Y., Zhu L., He J. (2022). Morphogenesis, Growth Cycle and Molecular Regulation of Hair Follicles. Front. Cell Dev. Biol..

[3] Schneider M.R., Schmidt-Ullrich R., Paus R. (2009). The Hair Follicle as a Dynamic Miniorgan. Curr. Biol..

[4] Wang B., Liu X.M., Liu Z.N., Wang Y., Han X., Lian A.B., Mu Y., Jin M.H., Liu J.Y. (2020). Human hair follicle-derived mesenchymal Stem. cells: Isolation, expansion, and differentiation. World J. Stem. Cells..

[5] Buffoli B., Rinaldi F., Labanca M., Sorbellini E., Trink A., Guanziroli E., Rezzani R., Rodella L.F. (2014). The human hair: From anatomy to physiology. Int. J. Dermatol..

[6] Piyaman P., Patchanee K., Oonjitti T., Ratanalekha R., Yodrabum N. (2020). Surgical anatomy of vascularized submental lymph node flap: Sharing arterial supply of lymph nodes with the skin and topographic relationship with anterior belly of digastric muscle. J. Surg. Oncol..

[7] Morgan B.A. (2014). The Dermal Papilla: An Instructive Niche for Epithelial Stem. and Progenitor Cells in Development and Regeneration of the Hair Follicle. CSH Perspect Med..

[8] Zhang B., Chen T. (2024). Local and systemic mechanisms that control the hair follicle Stem. cell niche. Nat. Rev. Mol. Cell Biol..

[9] Ji S.F., Zhu Z.Y., Sun X.Y., Fu X.B. (2021). Functional hair follicle regeneration: An updated review. Signal Transduct. Target Ther..

[10] Alzoabi N.M., Alsharif H.S.r., Alawami A.M., Habarah H.H., Alhawaj H.A., Bin Rubaian N., Alqahtani J.M. (2023). Assessing the Impact of Alopecia on Quality of Life, Depression, and Self-Esteem in Saudi Arabia. Cureus J. Med. Sci..

[11] Gokce N., Basgoz N., Kenanoglu S., Akalin H., Ozkul Y., Ergoren M.C., Beccari T., Bertelli M., Dundar M. (2022). An overview of the genetic aspects of hair loss and its connection with nutrition. J. Prev. Med. Hyg..

[12] Hasan R., Juma H., Eid F.A., Alaswad H.A., Ali W.M., Aladraj F.J. (2022). Effects of Hormones and Endocrine Disorders on Hair Growth. Cureus.

[13] Zeberkiewicz M., Rudnicka L., Malejczyk J. (2020). Immunology of alopecia areata. Cent. Eur. J. Immunol..

[14] Natarelli N., Gahoonia N., Sivamani R.K. (2023). Integrative and Mechanistic Approach to the Hair Growth Cycle and Hair Loss. J. Clin. Med..

[15] Cash T.F. (2001). The psychology of hair loss and its implications for patient care. Clin. Dermatol..

[16] Houschyar K.S., Borrelli M.R., Tapking C., Popp D., Puladi B., Ooms M., Chelliah M.P., Rein S., Pförringer D., Thor D. (2020). Molecular Mechanisms of Hair Growth and Regeneration: Current Understanding and Novel Paradigms. Dermatology.

[17] Mulinari-Brenner F., Bergfeld W.F. (2003). Hair loss: Diagnosis and management. Cleve Clin. J. Med..

[18] Gupta M., Mysore V. (2016). Classifications of Patterned Hair Loss: A Review. J. Cutan Aesthet Surg..

[19] Cardoso C.O., Tolentino S., Gratieri T., Cunha-Filho M., Lopez R.F.V., Gelfuso G.M. (2021). Topical Treatment for Scarring and Non-Scarring Alopecia: An Overview of the Current Evidence. Clin. Cosmet. Investig. Dermatol..

[20] Olsen E.A., Whiting D., Bergfeld W., Miller J., Hordinsky M., Wanser R., Zhang P., Kohut B. (2007). A multicenter, randomized, placebo-controlled, double-blind clinical trial of a novel formulation of 5% minoxidil topical foam versus placebo in the treatment of androgenetic alopecia in men. J. Am. Acad. Dermatol..

[21] Modha J.D., Pathania Y.S. (2022). Comprehensive review of oral minoxidil in alopecia. J. Cosmet. Dermatol..

[22] Gupta A.K., Talukder M., Venkataraman M., Bamimore M.A. (2022). Minoxidil: A comprehensive review. J. Dermatol. Treat..

[23] Messenger A.G., Rundegren J. (2004). Minoxidil: Mechanisms of action on hair growth. Br. J. Dermatol..

[24] Chislett B., Chen D., Perera M.L., Chung E., Bolton D., Qu L.G. (2023). 5-alpha reductase inhibitors use in prostatic disease and beyond. Transl. Androl. Urol..

[25] Zhou Z., Song S., Gao Z., Wu J., Ma J., Cui Y. (2019). The efficacy and safety of dutasteride compared with finasteride in treating men with androgenetic alopecia: A systematic review and meta-analysis. Clin. Interv. Aging..

[26] Escamilla-Cruz M., Magana M., Escandon-Perez S., Bello-Chavolla O.Y. (2023). Use of 5-Alpha Reductase Inhibitors in Dermatology: A Narrative Review. Dermatol Ther..

[27] Shimizu Y., Ntege E.H., Sunami H., Inoue Y. (2022). Regenerative medicine strategies for hair growth and regeneration: A narrative review of literature. Regen. Ther..

[28] Kesika P., Sivamaruthi B.S., Thangaleela S., Bharathi M., Chaiyasut C. (2023). Role and Mechanisms of Phytochemicals in Hair Growth and Health. Pharmaceuticals.

[29] Kaur S., Samota M.K., Choudhary M., Choudhary M., Pandey A.K., Sharma A., Thakur J. (2022). How do plants defend themselves against pathogens-Biochemical mechanisms and genetic interventions. Physiol. Mol. Biol. Plants.

[30] Al-Khayri J.M., Rashmi R., Toppo V., Chole P.B., Banadka A., Sudheer W.N., Nagella P., Shehata W.F., Al-Mssallem M.Q., Alessa F.M. (2023). Plant Secondary Metabolites: The Weapons for Biotic Stress Management. Metabolites.

[31] Nasim N., Sandeep I.S., Mohanty S. (2022). Plant-derived natural products for drug discovery: Current approaches and prospects. Nucleus.

[32] Boo Y.C. (2020). Emerging Strategies to Protect the Skin from Ultraviolet Rays Using Plant-Derived Materials. Antioxidants.

[33] Boo Y.C. (2019). Can Plant Phenolic Compounds Protect the Skin from Airborne Particulate Matter?. Antioxidants.

[34] Sitarek P., Kowalczyk T., Wieezfinska J., Merecz-Sadowska A., Górski K., Sliwinski T., Skala E. (2020). Plant Extracts as a Natural Source of Bioactive Compounds and Potential Remedy for the Treatment of Certain Skin Diseases. Curr. Pharm. Design..

[35] Boo Y.C. (2024). Insights into How Plant-Derived Extracts and Compounds Can Help in the Prevention and Treatment of Keloid Disease: Established and Emerging Therapeutic Targets. Int. J. Mol. Sci..

[36] Soe Z.C., Ei Z.Z., Visuttijai K., Chanvorachote P. (2023). Potential Natural Products Regulation of Molecular Signaling Pathway in Dermal Papilla Stem. Cells. Molecules.

[37] Daniels G., Akram S., Westgate G.E., Tamburic S. (2019). Can plant-derived phytochemicals provide symptom relief for hair loss? A critical review. Int. J. Cosmetic Sci..

[38] Rho S.S., Park S.J., Hwang S.L., Lee M.H., Kim C.D., Lee I.H., Chang S.Y., Rang M.J. (2005). The hair growth promoting effect of Asiasari radix extract and its molecular regulation. J. Dermatol. Sci..

[39] Kawano M., Han J., Kchouk M.E., Isoda H. (2009). Hair growth regulation by the extract of aromatic plant Erica multiflora. J. Nat. Med..

[40] Park P.J., Moon B.S., Lee S.H., Kim S.N., Kim A.R., Kim H.J., Park W.S., Choi K.Y., Cho E.G., Lee T.R. (2012). Hair growth-promoting effect of Aconiti Ciliare Tuber extract mediated by the activation of Wnt/beta-catenin signaling. Life Sci..

[41] Junlatat J., Sripanidkulchai B. (2014). Hair growth-promoting effect of Carthamus tinctorius floret extract. Phytother. Res..

[42] Kim E.J., Choi J.Y., Park B.C., Lee B.H. (2014). Platycarya strobilacea S. et Z. Extract Has a High Antioxidant Capacity and Exhibits Hair Growth-promoting Effects in Male C57BL/6 Mice. Prev. Nutr. Food Sci..

[43] Park G.H., Park K.Y., Cho H.I., Lee S.M., Han J.S., Won C.H., Chang S.E., Lee M.W., Choi J.H., Moon K.C. (2015). Red ginseng extract promotes the hair growth in cultured human hair follicles. J. Med. Food..

[44] Lee H., Kim N.H., Yang H., Bae S.K., Heo Y., Choudhary I., Kwon Y.C., Byun J.K., Yim H.J., Noh B.S. (2016). The Hair Growth-Promoting Effect of Rumex japonicus Houtt. Extract. Evid. Based Complement. Alternat. Med..

[45] Yu J.Y., Gupta B., Park H.G., Son M., Jun J.H., Yong C.S., Kim J.A., Kim J.O. (2017). Preclinical and Clinical Studies Demonstrate That the Proprietary Herbal Extract DA-5512 Effectively Stimulates Hair Growth and Promotes Hair Health. Evid. Based Complement. Alternat. Med..

[46] Boisvert W.A., Yu M., Choi Y., Jeong G.H., Zhang Y.L., Cho S., Choi C., Lee S., Lee B.H. (2017). Hair growth-promoting effect of Geranium sibiricum extract in human dermal papilla cells and C57BL/6 mice. BMC Complement. Altern Med..

[47] Somsukskul I., De-Eknamkul W., Tengamnuay P. (2017). Effect of Orthosiphon stamineus plant extract on in vitro dermal papilla cell proliferation and ex vivo hair growth. Chulalongkorn Med. J..

[48] Wen T.C., Li Y.S., Rajamani K., Harn H.J., Lin S.Z., Chiou T.W. (2018). Effect of Cinnamomum osmophloeum Kanehira Leaf Aqueous Extract on Dermal Papilla Cell Proliferation and Hair Growth. Cell Transplant..

[49] Kim J., Shin J.Y., Choi Y.H., Jang M., Nam Y.J., Lee S.Y., Jeon J., Jin M.H., Lee S. (2019). Hair Growth Promoting Effect of Hottuynia cordata Extract in Cultured Human Hair Follicle Dermal Papilla Cells. Biol. Pharm. Bull..

[50] Kang M.G., Park D., Han H.Y., Shim H., Hong Y., Moon J., Yoon S., Kwon B. (2019). RE-ORGA, a Korean Herb Extract, Can Prevent Hair Loss Induced by Dihydrotestosterone in Human Dermal Papilla Cells. Ann. Dermatol..

[51] Shin J.Y., Choi Y.H., Kim J., Park S.Y., Nam Y.J., Lee S.Y., Jeon J.H., Jin M.H., Lee S. (2020). Polygonum multiflorum extract support hair growth by elongating anagen phase and abrogating the effect of androgen in cultured human dermal papilla cells. BMC Complement. Med. Ther..

[52] Jin G.R., Zhang Y.L., Yap J., Boisvert W.A., Lee B.H. (2020). Hair growth potential of Salvia plebeia extract and its associated mechanisms. Pharm. Biol..

[53] Yamada N., Miki K., Yamaguchi Y., Takauji Y., Yamakami Y., Hossain M.N., Ayusawa D., Fujii M. (2020). Extract of Plumbago zeylanica enhances the growth of hair follicle dermal papilla cells with down-regulation of 5α-reductase type II. J. Cosmet. Dermatol..

[54] Serruya R., Maor Y. (2021). Hair growth-promotion effects at the cellular level and antioxidant activity of the plant-based extract Phyllotex™. Heliyon.

[55] Park H.J., Jin G.R., Jung J.H., Hwang S.B., Lee S.H., Lee B.H. (2021). Hair Growth Promotion Effect of Nelumbinis Semen Extract with High Antioxidant Activity. Evid. Based Complement. Alternat. Med..

[56] Ramadhani F.J., Bak D.H., Kang S.H., Park C.H., Park S.H., Chung B.Y., Bai H.W. (2022). The effects of centipedegrass extract on hair growth via promotion of anagen inductive activity. PLoS ONE.

[57] Ruksiriwanich W., Khantham C., Muangsanguan A., Chittasupho C., Rachtanapun P., Jantanasakulwong K., Phimolsiripol Y., Sommano S.R., Sringarm K., Ferrer E. (2022). Phytochemical Constitution, Anti-Inflammation, Anti-Androgen, and Hair Growth-Promoting Potential of Shallot (*Allium ascalonicum* L.) Extract. Plants.

[58] Wang J., Shen H., Chen T., Ma L. (2022). Hair growth-promoting effects of Camellia seed cake extract in human dermal papilla cells and C57BL/6 mice. J. Cosmet. Dermatol..

[59] Lee H., Kim H., Kim J.H., Park S.D., Shim J.J., Lee J.L. (2022). Lactobacillus paracasei HY7015 and Lycopus lucidus Turcz. Extract Promotes Human Dermal Papilla Cell Cytoprotective Effect and Hair Regrowth Rate in C57BL/6 Mice. Molecules.

[60] Tan Y.F., Koay Y.S., Zulkifli R.M., Hamid M.A. (2022). In Vitro hair growth and hair tanning activities of mangosteen pericarp extract on hair dermal papilla cells. J. Herb. Med..

[61] You J., Woo J., Roh K.B., Ryu D., Jang Y., Cho E., Park D., Jung E. (2023). Assessment of the anti-hair loss potential of Camellia japonica fruit shell extract in vitro. Int. J. Cosmet. Sci..

[62] Liang C.H., Lin Y.H., Lin Y.K., Chiang C.F. (2023). Hair growth-promotion effects and antioxidant activity of the banana flower extract HappyAngel?: Double-blind, placebo-controlled trial. Food Sci. Hum. Well..

[63] Iwabuchi T., Ogura K., Hagiwara K., Ueno S., Kitamura H., Yamanishi H., Tsunekawa Y., Kiso A. (2024). Ginsenosides in Panax ginseng Extract Promote Anagen Transition by Suppressing BMP4 Expression and Promote Human Hair Growth by Stimulating Follicle-Cell Proliferation. Biol. Pharm. Bull..

[64] Liu X., Lv X., Ji T., Hu H., Chang L. (2024). Gynostemma pentaphyllum Makino extract induces hair growth and exhibits an anti-graying effect via multiple mechanisms. J. Cosmet. Dermatol..

[65] You J., Woo J., Roh K.B., Jeon K., Jang Y., Choi S.A., Ryu D., Cho E., Park D., Lee J. (2024). Evaluation of efficacy of Silybum marianum flower extract on the mitigating hair loss in vitro and in vivo. J. Cosmet. Dermatol..

[66] Luo Z., Zhang X. (2022). Brassica oleracea extract, glucosinlates, and sulforaphane promote hair growth in vitro and ex vivo. J. Cosmet. Dermatol..

[67] Ma L., Shen H., Fang C., Chen T., Wang J. (2022). Camellia Seed Cake Extract Supports Hair Growth by Abrogating the Effect of Dihydrotestosterone in Cultured Human Dermal Papilla Cells. Molecules.

[68] Woo M.J., Kang H.Y., Paik S.J., Choi H.J., Uddin S., Lee S., Kim S.Y., Choi S., Jung S.K. (2023). The In Vivo and In Vitro Effects of *Terminalia bellirica* (Gaertn.) Roxb. Fruit Extract on Testosterone-Induced Hair Loss. J. Microbiol. Biotechnol..

[69] Bullwinkel J., Baron-Lühr B., Lüdemann A., Wohlenberg C., Gerdes J., Scholzen T. (2006). Ki-67 protein is associated with ribosomal RNA transcription in quiescent and proliferating cells. J. Cell Physiol..

[70] Pi L.Q., Lee W.S., Min S.H. (2016). Hot water extract of oriental melon leaf promotes hair growth and prolongs anagen hair cycle: In vivo and in vitro evaluation. Food Sci. Biotechnol..

[71] Li J.J., Li Z., Gu L.J., Choi K.J., Kim D.S., Kim H.K., Sung C.K. (2018). The promotion of hair regrowth by topical application of a Perilla frutescens extract through increased cell viability and antagonism of testosterone and dihydrotestosterone. J. Nat. Med..

[72] Hashimoto M., Kawai Y., Masutani T., Tanaka K., Ito K., Iddamalgoda A. (2022). Effects of watercress extract fraction on R-spondin 1-mediated growth of human hair. Int. J. Cosmetic. Sci..

[73] Fard S.G., Shamsabadi F.T., Emadi M., Meng G.Y., Muhammad K., Mohamed S. (2011). Ethanolic Extract of Eucheuma cottonii Promotes in vivo Hair Growth and Wound Healing. J. Anim. Vet. Adv..

[74] Rajan P., Natraj P., Kim N.H., Kim J.H., Choi H.J., Han C.H. (2023). Effects of Cudrania tricuspidata and Sargassum fusiforme extracts on hair growth in C57BL/6 mice. Lab. Anim. Res..

[75] Shimizu K., Kondo R., Sakai K., Shoyama Y., Sato H., Ueno T. (2000). Steroid 5alpha-reductase inhibitory activity and hair regrowth effects of an extract from Boehmeria nipononivea. Biosci. Biotechnol. Biochem..

[76] Roh S.S., Kim C.D., Lee M.H., Hwang S.L., Rang M.J., Yoon Y.K. (2002). The hair growth promoting effect of Sophora flavescens extract and its molecular regulation. J. Dermatol. Sci..

[77] Datta K., Singh A.T., Mukherjee A., Bhat B., Ramesh B., Burman A.C. (2009). Eclipta alba extract with potential for hair growth promoting activity. J. Ethnopharmacol..

[78] Murkute A.V., Sahu M.S., Mali P.Y., Rangari V.D. (2010). Development and evaluation of formulations of microbial biotransforMed. extract of tobacco leaves for hair growth potential. Pharmacogn. Res..

[79] Park H.J., Zhang N., Park D.K. (2011). Topical application of Polygonum multiflorum extract induces hair growth of resting hair follicles through upregulating Shh and β-catenin expression in C57BL/6 mice. J. Ethnopharmacol..

[80] Upadhyay S., Ghosh A.K., Singh V. (2012). Hair Growth Promotant Activity of Petroleum Ether Root Extract of *Glycyrrhiza Glabra* L (Fabaceae) in Female Rats. Trop. J. Pharm. Res..

[81] Sandhya S., Chandrasekhar J., Vinod K., Banji D. (2012). Potentiality of aqueous leaf extract of *Trichosanthes cucumerina* Linn. on hair growth promotion in Wistar albino rats. Indian J. Nat. Prod. Resour..

[82] Hou I.C., Oi Y., Fujita H., Yano Y., Fukami H., Yoshikawa M. (2013). A hair growth-promoting effect of Chinese black tea extract in mice. Biosci. Biotechnol. Biochem..

[83] Zhang N.N., Park D.K., Park H.J. (2013). Hair growth-promoting activity of hot water extract of *Thuja orientalis*. BMC Complement. Altern Med..

[84] Murata K., Noguchi K., Kondo M., Onishi M., Watanabe N., Okamura K., Matsuda H. (2013). Promotion of hair growth by Rosmarinus officinalis leaf extract. Phytother Res..

[85] Choi J.S., Jung S.K., Jeon M.H., Moon J.N., Moon W.S., Ji Y.H., Choi I.S., Wook Son S. (2013). Effects of *Lycopersicon esculentum* extract on hair growth and alopecia prevention. J. Cosmet. Sci..

[86] Choi J.S., Jeon M.H., Moon W.S., Moon J.N., Cheon E.J., Kim J.W., Jung S.K., Ji Y.H., Son S.W., Kim M.R. (2014). In vivo hair growth-promoting effect of rice bran extract prepared by supercritical carbon dioxide fluid. Biol. Pharm. Bull..

[87] Li Z., Li J., Gu L., Begum S., Wang Y., Sun B., Lee M., Sung C. (2014). Chrysanthemum zawadskii extract induces hair growth by stimulating the proliferation and differentiation of hair matrix. Int. J. Mol. Med..

[88] Punasiya R., Verma R., Pillai S. (2014). In vitro hair growth promoting activity of various leaves extract of *Hibiscus syriacus* L. on albino rats. Int. J. Pharm. Life Sci..

[89] Rozianoor M.W., Nadia M.F., Dzulsuhaimi D. (2014). In vivo evaluation of hair growth potential of Stachytarpheta jamaicensis ethanolic leaves extract on Sprague Dawley rats. Nat. Prod. Ann. Indian J..

[90] Begum S., Gu L.J., Lee M.R., Li Z., Li J.J., Hossain M.J., Wang Y.B., Sung C.K. (2015). In vivo hair growth-stimulating effect of medicinal plant extract on BALB/c nude mice. Pharm. Biol..

[91] Park S.O., Park B.S., Noh G.Y. (2015). Action mechanism of natural plant extracts for hair loss prevention and hair growth promotion in C57BL/6 mice. Int. J. Pharmacol..

[92] Mondal S., Ghosh D., Ganapaty S., Sushrutha M. (2016). Preliminary phytochemical analysis and evaluation of hair growth stimulating potential of ethanol extract from L. (Asteraceae) leaves in Wistar albino Eclipta alba rats. Asian J. Pharm. Pharmacol..

[93] Imtiaz F., Islam M., Saeed H., Saleem B., Asghar M., Saleem Z. (2017). Impact of Trigonella foenum-graecum Leaves Extract on Mice Hair Growth Leaves Extract on Mice Hair Growth. Pak. J. Zool..

[94] Zhu H.L., Gao Y.H., Yang J.Q., Li J.B., Gao J. (2018). Serenoa repens extracts promote hair regeneration and repair of hair loss mouse models by activating TGF-β and mitochondrial signaling pathway. Eur. Rev. Med. Pharmacol. Sci..

[95] Nanashima N., Horie K. (2019). Blackcurrant Extract with Phytoestrogen Activity Alleviates Hair Loss in Ovariectomized Rats. Molecules.

[96] Wang Q., Wang Y.X., Pang S.L., Zhou J., Cai J., Shang J. (2019). Alcohol extract from *Vernonia anthelmintica* willd (L.) seed counteracts stress-induced murine hair follicle growth inhibition. BMC Complement. Altern. Med..

[97] Amin J., Djajadisastra J., Syafhan N.F., Simamora E.L.P., Wulandari K. (2019). Green tea [*Camellia sinensis* (L.) kuntze] leaves extract and hibiscus (*Hibiscus tilliaceus* L.) leaves extract as topical hair growth promoter in microemulsion. Agr. Nat. Resour..

[98] Lee T.K., Kim B., Kim D.W., Ahn J.H., Sim H., Lee J.C., Yang G.E., Her Y., Park J.H., Kim H.S. (2020). Effects of Decursin and Angelica gigas Nakai Root Extract on Hair Growth in Mouse Dorsal Skin via Regulating Inflammatory Cytokines. Molecules.

[99] Ahn J.H., Park Y.E., Kim B., Park C.W., Sim T.H., Lee T.K., Lee J.C., Park J.H., Kim J.D., Lee H.S. (2020). Hair Growth is Promoted in Mouse Dorsal Skin by a Mixture of *Platycladus orientalis* (L.) Franco Leaf Extract and Alpha-Terpineol by Increasing Growth Factors and wnt3/β-Catenin. Nat. Prod Commun..

[100] Rose L.C., Rusdi N.N.S., Asari A., Abd Wahid M.E., Suhaimi H. (2020). Potential hair growth of crude extract from Hibiscus rosa-sinensis Linn. Arch. Pharm. Pract..

[101] Putra I.B., Jusuf N.K., Sumantri I.B. (2020). The Potency of Hibiscus rosa-sinensis Linn. Leaves Ethanol Extract as Hair Growth. Open Access Maced J. Med. Sci..

[102] Chavan R.R., Bhinge S.D., Bhutkar M.A., Randive D.S., Wadkar G.H., Todkar S.S. (2021). In vivo and in vitro hair growth-promoting effect of silver and iron nanoparticles synthesized via Blumea eriantha DC plant extract. J. Cosmet. Dermatol..

[103] Sakib S.A., Tareq A.M., Islam A., Rakib A., Islam M.N., Uddin M.A., Rahman M.M., Seidel V., Emran T.B. (2021). Anti-Inflammatory, Thrombolytic and Hair-Growth Promoting Activity of the n-Hexane Fraction of the Methanol Extract of Leea indica Leaves. Plants.

[104] Bhinge S.D., Bhutkar M.A., Randive D.S., Wadkar G.H., Todkar S.S., Savali A.S., Chittapurkar H.R. (2021). Screening of hair growth promoting activity of *Punica granatum* L. (pomegranate) leaves extracts and its potential to exhibit antidandruff and anti-lice effect. Heliyon.

[105] Madhunithya E., Venkatesh G., Shyamala G., Manjari V., Ramesh S., Karuppaiah A., Sankar V. (2021). Development of ethosome comprising combined herbal extracts and its effect on hair growth. Adv. Tradit. Med..

[106] Nursiyah N., Saputri R.K., Al-Bari A. (2021). Hair Growth Activity Test of Hair Tonic That Contain Green Tea Leaf Extract, Celery Leaf Extract and Combination of Green Tea Leaf and Celery Leaf Extract. Ad-Dawaa: J. Pharm. Sci..

[107] Rahmi I.A., Mun’im A., Jufri M. (2021). Formulation and evaluation of phytosome lotion from Nothopanax scutellarium leaf extract for hair growth. Int. J. Appl Pharm..

[108] Her Y., Lee T.K., Sim H., Lee J.C., Kim D.W., Choi S.Y., Hong J.K., Lee J.W., Kim J.D., Won M.H. (2022). Pinus thunbergii bark extract rich in flavonoids promotes hair growth in dorsal skin by regulating inflammatory cytokines and increasing growth factors in mice. Mol. Med. Rep..

[109] Bhinge S.D., Jadhav N.R., Randive D.S., Bhutkar M.A., Chavan R., Kumbhar B.V. (2022). Isolation and identification of hair growth potential fraction from active plant extract of Blumea eriantha DC grown in Western Ghat of India: In silico study. J. Ayurveda Integr. Med..

[110] Jung H., Jung D.M., Lee S.S., Kim E.M., Yoon K., Kim K.K. (2022). Mangifera Indica leaf extracts promote hair growth via activation of Wnt signaling pathway in human dermal papilla cells. Anim. Cells Syst..

[111] Gunawan E., Mochta Mano D.F., Dewi K., Pratiwi R.D. (2022). Hair Growth Test in Male Rabbits (Oryctolagus cuniculus) With Variations in The Concentration of Ethanol Extract Terentang (Campnosperma brevipetiolatum Volkens) Stem. Barks. J. Adv. Pharm. Pract..

[112] Leny L., Fitri K., Lase Y.K., Hafiz I., Iskandar B. (2022). Formulation of Hair Tonic from Ethanol Extract of Sea Hibiscus (*Hibiscus tileaceus* L.) Leaves in Promoting Hair Growth on Guinea Pig (*Cavia porcellus*). J. Drug Deliv. Ther..

[113] Shibato J., Takenoya F., Kimura A., Min C.W., Yamashita M., Gupta R., Kim S.T., Rakwal R., Shioda S. (2023). Examining the Effect of Notocactus ottonis Cold Vacuum Isolated Plant Cell Extract on Hair Growth in C57BL/6 Mice Using a Combination of Physiological and OMICS Analyses. Molecules.

[114] Dangi I., Sahu M., Verma L., Banweer J. (2023). Hair growth stimulating effect and phytochemical evaluation hydoalcoholic extract of Carica papaya leaves. World J. Pharm. Res..

[115] Tendri Adjeng A.N., Puspita Sarry E., Muhammad Ali N.F., Suryani S. (2023). Hair Growth-Promoting Activity of Hair Tonic containing Delipidated Ethanol Extract of *Capsicum frutescens* L. Leaves on Male Rabbit (*Oryctolagus cuniculus*). Res. J. Pharm. Technol..

[116] Lailiyah M. (2023). Hair Growth Cream Formulation from Shoe Flower Leaf Ethanol Extract (*Hibiscus rosa-sinensis* L.) As a Hair Grower in Rabbit (*Oryctolagus cuniculus*). J. Eduhealth.

[117] Müller-Röver S., Handjiski B., van der Veen C., Eichmüller S., Foitzik K., McKay I.A., Stenn K.S., Paus R. (2001). A comprehensive guide for the accurate classification of murine hair follicles in distinct hair cycle stages. J. Investig. Dermatol..

[118] Baek J.H., Lee S.Y., Yoo M., Park W.S., Lee S.J., Boo Y.C., Koh J.S. (2011). Effects of a new mild shampoo for preventing hair loss in Asian by a simple hand-held phototrichogram technique. Int. J. Cosmet. Sci..

[119] Kovacevic M., McCoy J., Goren A., Situm M., Stanimirovic A., Liu W., Tan Y., Vano-Galvan S., Shapiro J., Sinclair R. (2019). Novel shampoo reduces hair shedding by contracting the arrector pili muscle via the trace amine-associated receptor. J. Cosmet. Dermatol..

[120] Vicente R.A., Leite e Silva V.R., Baby A.R., Velasco M.V., Bedin V. (2009). Double-blind, randomized, placebo-controlled trial of a cream containing the *Stryphnodendron adstringens* (Martius) Coville bark extract for suppressing terminal hair growth. J. Eur. Acad. Dermatol. Venereol..

[121] Choi J.S., Park J.B., Moon W.S., Moon J.N., Son S.W., Kim M.R. (2015). Safety and Efficacy of Rice Bran Supercritical CO_2_ Extract for Hair Growth in Androgenic Alopecia: A 16-Week Double-Blind Randomized Controlled Trial. Biol. Pharm. Bull..

[122] Srivilai J., Nontakhot K., Nutuan T., Waranuch N., Khorana N., Wisuthiprot W., Scholfield C.N., Champachaisri K., Ingkaninan K. (2018). Sesquiterpene-Enriched Extract of Curcuma aeruginosa Roxb. Retards Axillary Hair Growth: A Randomised, Placebo-Controlled, Double-Blind Study. Skin Pharmacol. Physiol..

[123] Pekmezci E., Dundar C., Turkoglu M. (2018). A proprietary herbal extract against hair loss in androgenetic alopecia and telogen effluvium: A placebo-controlled, single-blind, clinical-instrumental study. Acta Dermatovenerol. Alp. Pannonica Adriat..

[124] Choi H.C., Nam G.W., Jeong N.H., Choi B.Y. (2019). Hair Growth Promotion by Extracts of Inula Helenium and Caesalpinia Sappan Bark in Patients with Androgenetic Alopecia: A Pre-clinical Study Using Phototrichogram Analysis. Cosmetics.

[125] Kim B.H., Lee W.Y., Trinh T.A., Pyo J.S., Lee S., Kim C.E., Lee D.H., Park E.S., Kang K.S. (2020). Hair Growth Effect of Emulsion Extracted Brevilin A, a JAK3 Inhibitor, from Centipeda minima. Processes.

[126] Ham S., Lee Y.I., Kim I.A., Suk J., Jung I., Jeong J.M., Lee J.H. (2023). Efficacy and safety of persimmon leaf formulated with green tea and sophora fruit extracts (BLH308) on hair growth: A randomized, double-blind, placebo-controlled clinical trial. Skin Res. Technol..

[127] Sharifi-Rad M., Kumar N.V.A., Zucca P., Varoni E.M., Dini L., Panzarini E., Rajkovic J., Fokou P.V.T., Azzini E., Peluso I. (2020). Lifestyle, Oxidative Stress, and Antioxidants: Back and Forth in the Pathophysiology of Chronic Diseases. Front. Physiol..

[128] Trüeb R.M. (2021). Oxidative stress and its impact on skin, scalp and hair. Int. J. Cosmet. Sci..

[129] Trüeb R.M. (2015). The impact of oxidative stress on hair. Int. J. Cosmetic Sci..

[130] Trüeb R.M., Henry J.P., Davis M.G., Schwartz J.R. (2018). Scalp Condition Impacts Hair Growth and Retention via Oxidative Stress. Int. J. Trichol..

[131] Zhai X., Gong M., Peng Y., Yang D. (2021). Effects of UV Induced-Photoaging on the Hair Follicle Cycle of C57BL6/J Mice. Clin. Cosmet. Investig. Dermatol..

[132] Jun M.S., Kwack M.H., Kim M.K., Kim J.C., Sung Y.K. (2020). Particulate Matters Induce Apoptosis in Human Hair Follicular Keratinocytes. Ann. Dermatol..

[133] Choi D., Choi J.Y., Lee J.B., Yun S.J., Moon B.K., Ahn Y.G., Lee S.Y., Lee S.C. (2023). Protective Activity against Oxidative Stress in Dermal Papillae with Extracted Herbal Essential Oils. Appl. Sci..

[134] Fernández E., Martínez-Teipel B., Armengol R., Barba C., Coderch L. (2012). Efficacy of antioxidants in human hair. J. Photoch. Photobiol. B..

[135] Muangsanguan A., Linsaenkart P., Chaitep T., Sangta J., Sommano S.R., Sringarm K., Arjin C., Rachtanapun P., Jantanasakulwong K., Phimolsiripol Y. (2023). Hair Growth Promotion and Anti-Hair Loss Effects of By-Products Arabica Coffee Pulp Extracts Using Supercritical Fluid Extraction. Foods.

[136] Erekat N.S. (2022). ProgramMed. Cell Death in Diabetic Nephropathy: A Review of Apoptosis, Autophagy, and Necroptosis. Med. Sci. Monitor..

[137] Jan R., Chaudhry G.E. (2019). Understanding Apoptosis and Apoptotic Pathways Targeted Cancer Therapeutics. Adv. Pharm. Bull..

[138] Lee E.W., Seo J., Jeong M., Lee S., Song J. (2012). The roles of FADD in extrinsic apoptosis and necroptosis. BMB Rep..

[139] Han Y.H., Wang Y., Lee S.J., Jin M.H., Sun H.N., Kwon T. (2023). Regulation of anoikis by extrinsic death receptor pathways. Cell Commun. Signal..

[140] Heilmann-Heimbach S., Hochfeld L.M., Henne S.K., Nothen M.M. (2020). Hormonal regulation in male androgenetic alopecia-Sex hormones and beyond: Evidence from recent genetic studies. Exp. Dermatol..

[141] Olsen E.A., Hordinsky M., Whiting D., Stough D., Hobbs S., Ellis M.L., Wilson T., Rittmaster R.S., Dutasteride Alopecia Research T. (2006). The importance of dual 5alpha-reductase inhibition in the treatment of male pattern hair loss: Results of a randomized placebo-controlled study of dutasteride versus finasteride. J. Am. Acad. Dermatol..

[142] Engeland K. (2022). Cell cycle regulation: p53-p21-RB signaling. Cell Death Differ..

[143] Duan J., Chen Z., Liu P., Zhang Z., Tong T. (2004). Wild-type p16INK4a suppresses cell growth, telomerase activity and DNA repair in human breast cancer MCF-7 cells. Int. J. Oncol..

[144] Trüeb R.M. (2018). Further Clinical Evidence for the Effect of IGF-1 on Hair Growth and Alopecia. Skin Appendage Disor..

[145] Li W., Man X.Y., Li C.M., Chen J.Q., Zhou J., Cai S.Q., Lu Z.F., Zheng M. (2012). VEGF induces proliferation of human hair follicle dermal papilla cells through VEGFR-2-mediated activation of ERK. Exp. Cell Res..

[146] Qi Y., Li M., Xu L., Chang Z., Shu X., Zhou L. (2016). Therapeutic role of human hepatocyte growth factor (HGF) in treating hair loss. PeerJ.

[147] Richardson G.D., Bazzi H., Fantauzzo K.A., Waters J.M., Crawford H., Hynd P., Christiano A.M., Jahoda C.A. (2009). KGF and EGF signalling block hair follicle induction and promote interfollicular epidermal fate in developing mouse skin. Development.

[148] Zeng H., Gu L., Maeda K. (2017). Evaluation of the effect of plant mixture ethanol extracts containing *Biota orientalis* L. extract on suppression of sebum in cultured sebocytes and on stimulation of growth of keratinocytes co-cultured with hair papilla cells. Cosmetics.

[149] Kim B.H., Lee M.J., Lee W.Y., Pyo J., Shin M.S., Hwang G.S., Shin D., Kim C.E., Park E.S., Kang K.S. (2021). Hair Growth Stimulation Effect of Centipeda minima Extract: Identification of Active Compounds and Anagen-Activating Signaling Pathways. Biomolecules.

[150] Yudushkin I. (2020). Control of Akt activity and substrate phosphorylation in cells. IUBMB Life.

[151] Shimura T., Kakuda S., Ochiai Y., Nakagawa H., Kuwahara Y., Takai Y., Kobayashi J., Komatsu K., Fukumoto M. (2010). Acquired radioresistance of human tumor cells by DNA-PK/AKT/GSK3β-mediated cyClin. D1 overexpression. Oncogene.

[152] Hua H., Zhang H.Y., Chen J.Z., Wang J., Liu J.Y., Jiang Y.F. (2021). Targeting Akt in cancer for precision therapy. J. Hematol. Oncol..

[153] Tikkanen R., Nikolic-Paterson D.J. (2019). Mitogen-Activated Protein Kinases: Functions in Signal Transduction and Human Diseases. Int. J. Mol. Sci..

[154] Plotnikov A., Zehorai E., Procaccia S., Seger R. (2011). The MAPK cascades: Signaling components, nuclear roles and mechanisms of nuclear translocation. Biochim. Biophys. Acta-Mol. Cell Res..

[155] Braicu C., Buse M., Busuioc C., Drula R., Gulei D., Raduly L., Rusu A., Irimie A., Atanasov A.G., Slaby O. (2019). A Comprehensive Review on MAPK: A Promising Therapeutic Target in Cancer. Cancers.

[156] Liu J.Q., Xiao Q., Xiao J.N., Niu C.X., Li Y.Y., Zhang X.J., Zhou Z.W., Shu G., Yin G. (2022). Wnt/β-catenin signalling: Function, biological mechanisms, and therapeutic opportunities. Signal Transduct. Target Ther..

[157] Shang S., Hua F., Hu Z.W. (2017). The regulation of β-catenin activity and function in cancer: Therapeutic opportunities. Oncotarget.

[158] Lyros O., Rafiee P., Nie L.H., Medda R., Jovanovic N., Schmidt J., Mackinnon A., Venu N., Shaker R. (2014). Dickkopf-1, the Wnt antagonist, is induced by acidic pH and mediates epithelial cellular senescence in human reflux esophagitis. Am. J. Physiol.-Gastrointest. Liver Physiol..

[159] Kwack M.H., Sung Y.K., Chung E.J., Im S.U., Ahn J.S., Kim M.K., Kim J.C. (2008). Dihydrotestosterone-inducible dickkopf 1 from balding dermal papilla cells causes apoptosis in follicular keratinocytes. J. Investig. Dermatol..

[160] Papukashvili D., Rcheulishvili N., Liu C., Xie F.F., Tyagi D., He Y.J., Wang P.G. (2021). Perspectives on miRNAs Targeting DKK1 for Developing Hair Regeneration Therapy. Cells.

[161] Jing J.J., Wu Z.X., Wang J.H., Luo G.W., Lin H.Y., Fan Y., Zhou C.C. (2023). Hedgehog signaling in tissue homeostasis, cancers, and targeted therapies. Signal Transduct. Target Ther..

[162] Rishikaysh P., Dev K., Diaz D., Qureshi W.M.S., Filip S., Mokry J. (2014). Signaling Involved in Hair Follicle Morphogenesis and Development. Int. J. Mol. Sci..

[163] Carballo G.B., Honorato J.R., de Lopes G.P.F., Spohr T.C.L.D.E. (2018). A highlight on Sonic hedgehog pathway. Cell Commun. Signal..

[164] Sabol M., Trnski D., Musani V., Ozretic P., Levanat S. (2018). Role of GLI Transcription Factors in Pathogenesis and Their Potential as New Therapeutic Targets. Int. J. Mol. Sci..

[165] Aashaq S., Batool A., Mir S.A., Beigh M.A., Andrabi K.I., Shah Z.A. (2022). TGF-beta signaling: A recap of SMAD-independent and SMAD-dependent pathways. J. Cell Physiol..

[166] Tie Y., Tang F., Peng D., Zhang Y., Shi H. (2022). TGF-beta signal transduction: Biology, function and therapy for diseases. Mol. Biomed..

[167] Wu M.R., Wu S.L., Chen W., Li Y.P. (2024). The roles and regulatory mechanisms of TGF-β and BMP signaling in bone and cartilage development, homeostasis and disease. Cell Res..

[168] Peng D., Fu M., Wang M., Wei Y., Wei X. (2022). Targeting TGF-beta signal transduction for fibrosis and cancer therapy. Mol. Cancer.

[169] Shin H., Yoo H.G., Inui S., Itami S., Kim I.G., Cho A.R., Lee D.H., Park W.S., Kwon O., Cho K.H. (2013). Induction of transforming growth factor-beta 1 by androgen is mediated by reactive oxygen species in hair follicle dermal papilla cells. BMB Rep..

[170] Wu P., Zhang Y., Xing Y., Xu W., Guo H., Deng F., Ma X., Li Y. (2019). The balance of Bmp6 and Wnt10b regulates the telogen-anagen transition of hair follicles. Cell Commun. Signal..

[171] Kim H.S., Kwon H.K., Lee D.H., Le T.N., Park H.J., Kim M.I. (2019). Poly(gamma-Glutamic Acid)/Chitosan Hydrogel Nanoparticles For Effective Preservation And Delivery Of Fermented Herbal Extract For Enlarging Hair Bulb And Enhancing Hair Growth. Int. J. Nanomed..

[172] Grymowicz M., Rudnicka E., Podfigurna A., Napierala P., Smolarczyk R., Smolarczyk K., Meczekalski B. (2020). Hormonal Effects on Hair Follicles. Int. J. Mol. Sci..

[173] Horesh E.J., Cheret J., Paus R. (2021). Growth Hormone and the Human Hair Follicle. Int. J. Mol. Sci..

[174] O’Sullivan J.D.B., Peters E.M.J., Amer Y., Atuluru P., Cheret J., Rosenberg A.M., Picard M., Paus R. (2022). The impact of perceived stress on the hair follicle: Towards solving a psychoneuroendocrine and neuroimmunological puzzle. Front. Neuroendocrinol..

